# Predicting suitable coastal habitat for sei whales, southern right whales and dolphins around the Falkland Islands

**DOI:** 10.1371/journal.pone.0244068

**Published:** 2020-12-23

**Authors:** Mick Baines, Caroline R. Weir

**Affiliations:** 1 Wildscope, El Cuarton, Tarifa, Cadiz, Spain; 2 Falklands Conservation, Jubilee Villas, Stanley, Falkland Islands; Wildlife Conservation Society Canada, CANADA

## Abstract

Species distribution models (SDMs) are valuable tools for describing the occurrence of species and predicting suitable habitats. This study used generalized additive models (GAMs) and MaxEnt models to predict the relative densities of four cetacean species (sei whale *Balaeanoptera borealis*, southern right whale *Eubalaena australis*, Peale’s dolphin *Lagenorhynchus australis*, and Commerson’s dolphin *Cephalorhynchus commersonii*) in neritic waters (≤100 m depth) around the Falkland Islands, using boat survey data collected over three seasons (2017–2019). The model predictor variables (PVs) included remotely sensed environmental variables (sea surface temperature, SST, and chlorophyll-*a* concentration) and static geographical variables (e.g. water depth, distance to shore, slope). The GAM results explained 35 to 41% of the total deviance for sei whale, combined sei whales and unidentified large baleen whales, and Commerson’s dolphins, but only 17% of the deviance for Peale’s dolphins. The MaxEnt models for all species had low to moderate discriminatory power. The relative density of sei whales increased with SST in both models, and their predicted distribution was widespread across the inner shelf which is consistent with the use of Falklands’ waters as a coastal summer feeding ground. Peale’s dolphins and Commerson’s dolphins were largely sympatric across the study area. However, the relative densities of Commerson’s dolphins were generally predicted to be higher in nearshore, semi-enclosed, waters compared with Peale’s dolphins, suggesting some habitat partitioning. The models for southern right whales performed poorly and the results were not considered meaningful, perhaps due to this species exhibiting fewer strong habitat preferences around the Falklands. The modelling results are applicable to marine spatial planning to identify where the occurrence of cetacean species and anthropogenic activities may most overlap. Additionally, the results can inform the process of delineating a potential Key Biodiversity Area for sei whales in the Falkland Islands.

## Introduction

The delineation of discrete spatial areas that support high species densities is a fundamental component of effective marine spatial planning (MSP), particularly when designating areas of marine biodiversity for protection and in mitigating potential conflicts between human activities and marine species. Regionally, a number of marine protected areas, special areas of conservation, ecologically or biologically significant areas, and important marine mammal areas, have been designated for cetacean species and communities, which help to focus management where it is most needed [1, 2]. Most such designations are based on the identification of nationally or regionally significant cetacean occurrence, incorporating factors including conservation status, distribution, habitat use, abundance and key life-cycle activities such as reproduction, migration and feeding. Additionally, a set of standardised global criteria and thresholds have been developed by the International Union for Conservation of Nature in order to define key biodiversity areas (KBAs), comprising sites that contribute significantly to the globally persistence of biodiversity [3].

A fundamental consideration for all types of protected area is ensuring that the spatial boundaries reflect the distribution of species both at the time of designation and in the future, in alignment with predicted environmental fluctuations (e.g. seasonal and annual variations, and longer-term climate change). Species distribution models (SDMs) are valuable tools for understanding species occurrence and for the identification of important habitats, and are increasingly used in MSP. They estimate the statistical relationships between spatial and temporal species presence and environmental factors, and generate models with some level of predictive power [4]. Model outputs have conservation applications including predicting species occurrence in areas of unsurveyed habitat [5], predicting where species may overlap with anthropogenic activities [6–8], and predicting changes in species distribution range in response to environmental factors [9]. Cetacean occurrence is usually modelled against a range of topographic (e.g. water depth, slope), physical (e.g. seabed substrate type, proximity to river mouths), and oceanographic (e.g. sea surface temperature, chlorophyll, fronts) variables [7, 8, 10–12]. Since cetacean distributions may primarily be driven by those of their prey, it is likely that such factors serve as proxies for spatio-temporal variation in prey density [13]; information on the latter is only rarely directly obtainable [14]. Geographical and environmental space can be treated as continuous or discrete among the different SDM methods [15]. This study applied two different SDM approaches to model areas of predicted suitable habitat for four cetacean species in neritic waters (≤100 m depth) around the Falkland Islands in the south-west Atlantic: (1) pooling of data into discrete spatial units (e.g. grid cells or line segments), in which the predicted occurrence was modelled as a function of environmental variables; and (2) presence-only point data that were modelled directly [15]. The latter approach is widely used when only presence data are available [16]. However, it is equally applicable to presence-absence datasets, where the absence data are used to account for sampling bias [17, 18] over a wide range of spatial scales [19, 20].

Boat surveys to assess the distribution and abundance of baleen whales, particularly sei whales (*Balaenoptera borealis*), have been carried out annually in the Falklands since 2017 [21], to investigate the feasibility of designating KBAs for whales in the region. We used a boat survey dataset collected over three whale seasons (2017–2019) to model the predicted relative density of two baleen whale and two dolphin species across inner shelf waters, in order to inform KBA designation and conservation management planning.

## Materials and methods

### Ethics statement

This study was authorised by the Falkland Islands Government (research licences R23.2016 and R11.2017).

### Study area

**Fig 1:**
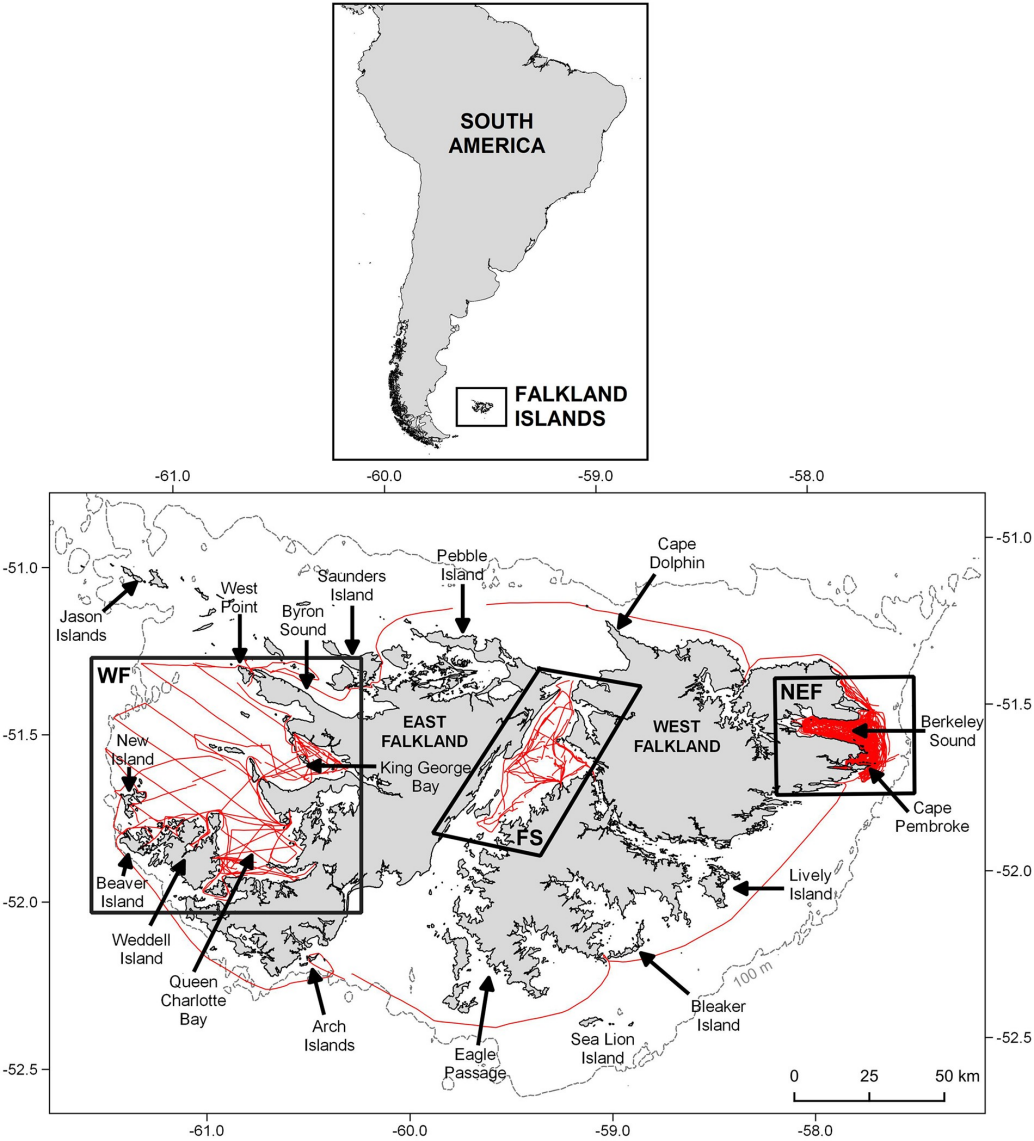
Location of the Falkland Islands study area. The three focal study sites between 2017 and 2019 comprise: (1) north-east coast of the Falklands (NEF); (2) the west coast of West Falkland (WF); and (3) the Falkland Sound (FS). Survey effort (all weather conditions) is shown in red. Falklands bathymetric and coastline shapefiles were accessed open source from the Information Management System (IMS) and GIS Data Centre in Stanley, Falkland Islands (available via https://www.south-atlantic-research.org/research/data-science).

The study area comprised shallow shelf waters around the Falkland Islands, which are located off the south-east coast of South America on a south-easterly extension of the Patagonian Shelf (Fig 1). Effort was focussed on three coastal sites: (1) Berkeley Sound and adjacent waters along the north-east coast of the Falklands (NEF); (2) the west coast of the Falklands (WF); and (3) Falkland Sound (FS), situated between East and West Falkland (Fig 1). In addition, survey data were collected during a circumnavigation of the Falklands during 2018. The sites were predominantly located in water depths of ≤60 m, but effort extended to depths of 100 m in WF, and during the circumnavigation.

### Data collection

Cetacean visual surveys were carried out during the austral summer (Jan–Feb), autumn (Mar–May), and winter (Jun–Aug). Effort occurred at NEF during 2017 (Feb–May) and 2019 (Jan–Aug), at WF during 2018 (Feb–Apr), and at FS during 2019 (Feb–Jun). The survey platforms comprised a 6.5 m rigid-hulled inflatable boat (RHIB; average survey speed of 21 km h^-1^ and eye height of ~2 m) in NEF and FS, and a 19.5 m yacht (average survey speed of 13 km h^-1^ and eye height of 5.1 m) in WF. The survey track generally comprised one of two standard routes in NEF, and three standardised routes in FS. However, the exact routes varied according to weather conditions, logistical constraints and cetacean encounters. In WF, coverage included a series of predetermined standardised transects conducted in passing mode, and non-systematic surveys variously conducted in both passing and closing mode (see Weir, 2018, for more details). Surveys only commenced in favourable conditions for detecting whales, defined as Beaufort sea states ≤4, swell heights ≤2 m, and good visibility (>5 km). Positional data were logged at 1 min intervals, and sea state, swell and visibility were recorded throughout the surveys and linked to each 1 min effort segment.

Effort status was logged as active search, cetacean encounter (when working with animals for photo-identification), or off effort. During active search effort, at least two observers located on the port and starboard sides of the vessel searched continuously for cetaceans by naked eye, each focussed on a 90° area from the bow to their beam. When cetaceans were sighted, the time, position, species, group size and behaviour were recorded. A distance (estimated by eye) and angle (measured using an angle board) were recorded for sightings that were observed in passing mode. For sightings recorded during closing mode, a position and time were recorded once the boat was in proximity to the animals.

### Data analysis

Survey data were projected into eastings and northings in units of metres (EPSG:32721—WGS 84 / UTM zone 21S), which allowed the creation of grids with equal cell dimensions. The choice of grid cell size involved a trade-off between precision, the ability to apply an appropriate error distribution, and attaining a meaningful output for biological conservation [22, 23]. The relationship between grid cell size and the proportion of sightings in which the observer and the sighting were located within the same grid cell, and were therefore associated with the same PVs, was investigated using a subset of sightings for which the estimated range and bearing from the platform were recorded at initial detection from which a position could be calculated. At a grid cell resolution of 4 km (the lowest resolution considered in this study, based on the available resolution of some predictor variables), only 68% of calculated sighting positions were located within the same grid cell as the observers. That proportion increased to 80% using a 7 km grid cell resolution (S1 Fig). The statistical package R (R Core Development Team, 2006) was used to create and populate grids, and the software package Quantum GIS (QGIS v.3.12) was used for mapping.

#### Data inclusion

Only periods of active search effort and associated sightings were included in the analysis. Although surveys only commenced in favourable weather, some periods of higher sea state (Beaufort >4), high swell (>2 m), or reduced visibility (<5 km) occurred during the surveys and were removed from the dataset prior to generalized additive modelling (GAM). Data collected in all weather conditions were included in the MaxEnt models.

Modelling was applied to cetacean species for which at least 80 on-effort sightings were available, comprising sei whale, southern right whale (*Eubalaena australis*), Peale’s dolphin (*Lagenorhynchus australis*), and Commerson’s dolphin (*Cephalorhynchus commersonii*). Both baleen whale species exhibit seasonality in coastal waters around the Falklands, including seasonal peaks of sei whales during summer and autumn [21], and of southern right whales during winter [24]. Initial assessments were carried out to determine whether using seasonally-limited datasets would improve the models. The use of a data subset collected between January and May did not alter the sei whale model results, and consequently the full temporal dataset was used. However, only data from May to August were used in the southern right whale modelling to avoid excessive zero inflation.

In addition to sei whales, a combined category comprising sei whales and unidentified large baleen whales (SEI–BAL) was modelled. That analysis was confined to data collected between January and April, since all large baleen whale sightings that were closed on were subsequently confirmed to comprise sei whales, and no other large baleen whale species were recorded during any surveys in those months [25, Falklands Conservation, unpublished data].

#### Predictor variables

The selection of predictor variables (PVs: Table 1) included in the models was determined by their potential biological relevance for describing cetacean habitat and their availability. PV data were extracted to raster grids using the R package *raster*; where the resolution or origin of grid cells differed between source and output, a mean function was applied. Sea surface temperature (SST) and chlorophyll-a concentration (Chl-*a*) were the only PVs to vary by month (Table 1). While monthly composite values do not provide fine-scale temporal information on habitat use, they do provide information on distribution in relation to longer-term local climate.

**Table 1:** Candidate predictor variables (PVs).

PV	Working name	Definition and method
***Water depth***	Depth	Water depth (m) extracted from the GEBCO grid (GEBCO Compilation Group, 2019).
***Aspect***	Aspect	Direction of the slope of the sea floor in degrees. Derived from the GEBCO grid using QGIS.
***Slope***	Slope	Degrees of slope of the seabed. Derived from the GEBCO grid using QGIS.
***Roughness***	Roughness	Mean variation in water depth (m) between adjacent pixels in the GEBCO raster, using QGIS.
***Distance to shore***	ShoreDist	Linear distance to the nearest shore, calculated using a custom function in R.
***Distance to kelp bed***	KelpDist	Linear distance to the nearest kelp bed. Kelp distribution was based on an image segmentation analysis of two Landsat 8 satellite imagery (2013 and 2014), carried out by Environment Systems Ltd (2014) for Premier Oil Ltd.
***Sea surface temperature***	SST	Sea surface temperature (°C) as monthly composites at 4 km resolution. Derived from the NASA OceanColor Web service (https://oceancolor.gsfc.nasa.gov/l3/)
***Chlorophyll-a concentration***	Chl-*a*	Chlorophyll-*a* concentration (mg m^-3^) as monthly composites at 4 km resolution. Derived from the NASA OceanColor Web service (https://oceancolor.gsfc.nasa.gov/l3/).
**Effort quantity**	EffortTotal	Segment length in km.
**Beaufort sea state**	SeaState	Beaufort sea state categories recorded during field survey effort.
**Swell height**	Swell	Swell height (m) recorded during field survey effort.
**Visibility**	Visibility	Visibility (km) recorded during field survey effort.
**Position**	X,Y	Eastings and northings projected at UTM zone 21S from position data collected on a hand-held GPS.
**Month**	Month	Month of the field study.
**Year**	Year	Year of the field study.

All PVs were tested for inclusion in the generalized additive models. PVs that were also included in the MaxEnt models are highlighted in italics.

Collinearity between PVs was examined using Pearson’s product-moment correlation tests in R. The only significantly correlated pair of PVs was ShoreDist and KelpDist (coefficient of 0.75). However, since the presence of kelp beds was considered to have ecological-relevance for several species, and because some areas close to the coast did not have kelp beds, both of those PVs (separately and in combination) were examined in the models and retained if they improved model fit.

#### Generalized additive models

The survey data (effort and associated sightings) and the PVs were compiled into data frames for generalized additive modelling (GAMs: [26]), based on grids of 4 km and 7 km resolution; the latter was subsequently determined to provide a better fit to the data for each species, and was therefore selected for the final modelling. Where consecutive effort segments occurred within the same 7 km grid cell with the same environmental variables, the data were pooled in order to minimise zero inflation. GAMs were carried out in R using the package *mgcv*. Initial exploratory models indicated that best fits were obtained with a negative-binomial distribution using the *nb()* function with log link designed for integrated estimation of parameter *theta* [27, 28]. For each transit of a grid cell, species presence data comprised the number of sighting events recorded (sightings) and the total number of individuals in those events (counts). For baleen whales, counts were modelled as the response variable. Separate models were tested for the dolphins using counts and sightings, and for both species the use of counts achieved a better fit.

Each PV entered the model as a smooth term (except Year and Month, which were included as factors), where the degree of smoothness was determined as part of the model fitting process [27]. Thin plate regression splines were used, since they allow smoothing with respect to any number of PVs and do not require ‘knot’ locations to be specified [27]. Plots of GAM smooth terms were examined and when they exhibited a tendency for over-fitting, the degrees of freedom of the respective smooth term was limited to 4. Statistical methods for comparing the goodness of fit between models are well established and GAM development generally involves a process of adding, subtracting or transforming variables in order to obtain the best fit by comparing measures such as AIC [29], GCV and UBRE scores [27]. PVs were only included in the final models when they improved model fit, and their contribution to the explained deviance was assessed using forward stepwise selection. Effort quantity (km) was incorporated into models as an offset. Sea state, swell and visibility were tested as parametric coefficients in all models and retained if they improved model fit. For some PVs in which the distribution of values was skewed, square root transformation was applied to spread values more evenly.

Predicted counts per grid cell, a metric equivalent to relative density, for sei whales, combined sei and unidentified large baleen whales, and the two dolphin species were made using a data frame with SST and chl-*a* values from March 2018, representing the seasonal peak in sei whale occurrence in the study area [21]. However, the predictions for southern right whales used SST and chl-*a* values from July 2019, corresponding with the middle of winter to reflect their known seasonality in the NEF.

To assess prediction error in the GAMs, a repeated cross-validation procedure was implemented. The data were randomly partitioned into training and testing sub-sets in the ratio of 0.75:0.25. GAMs were then run on both sub-sets, and the root mean squared difference between the two resulting vectors was calculated as the root-mean-square error (RMSE). This was repeated 10 times from which mean RMSE values were derived. RMSE is interpreted as how far, on average, the residuals are from zero [30].

#### MaxEnt

Predictive models of species occurrence were run in the software MaxEnt (version 3.4.1), which is based on a maximum entropy algorithm [31]. It is a point process model that allows each cetacean sighting position to be entered as a presence point and therefore potentially supports a finer resolution for the associated PV data. The latitude and longitude of each sighting were projected to an easting and northing using a QGIS function. The sighting positions used in MaxEnt were either recalculated from the boat position, angle and bearing (passing mode) or were the boat position when in closest proximity to the animals (closing mode). PV data were input as grids of 4 km resolution, as that was the finest resolution available for SST and Chl-*a* data. To account for sampling bias [32] a grid of normalised effort was constructed as:

$${Ε}_{t}\;=\frac{\varepsilon_{t}}{\sum_{t=1}^{n}\varepsilon_{t}}$$

Where E_*t*_ is the normalised effort value for cell *t* and ε_*t*_ is the effort in km for cell *t*.

20 replicate models were run, each with 20% randomly selected test points. The replicated run type was set to cross-validate and the output set to raw [33].

To perform many types of model evaluation, metrics of model fit are needed. Area under the receiver-operator curve (AUC) is most widely used in the MaxEnt literature [33]. AUC is a threshold independent measure of predictive accuracy based only on the ranking of locations, and is interpreted as the probability that a randomly chosen presence location is ranked higher than a randomly chosen background point. An AUC close to 1.0 indicates good power, while a value of ≤0.5 indicates that the model prediction is no better than random [34].

MaxEnt calculates two metrics for the relative importance of PVs in a model. The percent contribution is a measure of the increase in gain contributed by each variable at each step in the MaxEnt algorithm, converted to a percentage; the permutation importance is calculated by re-evaluating the final model by permuting each variable in turn and measuring the resulting decrease in AUC [31]. MaxEnt was set to the raw output format which outputs a relative occurrence rate (ROR), which for grid cells of equal area is equivalent to a measure of relative density.

#### Geographic scope of predictions

An important constraint applied to our models, was to avoid predicting distribution in habitat types that had not been sampled. Consequently, data from water depths exceeding 100 m were removed. This ensured that the final predicted distributions applied only to habitat for which survey effort was available against which to evaluate the models.

## Results

A total of 7,460 km of boat-based active search effort was collected in favourable conditions between 2017 and 2019, with 2,530 associated cetacean sightings (Table 2; S2–S5 Figs). Sei whales were recorded only between January and May, while southern right whales were recorded only between mid-May and August. Both dolphin species were observed in every survey month.

**Table 2:** Total search effort (km) in each year in favourable conditions (Beaufort sea state ≤4, swell height ≤2 m, visibility >5 km), and the number of associated Sightings (S) and Individual (I) recorded of each cetacean species.

Year	Effort (km)	Sei whale		Sei+large baleen whale*		Southern right whale		Peale’s dolphin		Commerson’s dolphin	
		S	I	S	I	S	I	S	I	S	I
**2017**	2,144	123	248	126	251	10	13	117	476	21	67
**2018**	2,132	364	642	892	1,316	0	0	163	559	117	399
**2019**	3,185	144	250	173	289	69	148	189	849	22	94
**Total**	7,460	631	1,140	1,191	1,856	79	161	469	1,884	160	560

*Unidentified, but almost certainly also sei whales [21, 25].

### Sei whale

The GAM for sei whales explained 34.7% of deviance and had a RMSE of 0.21 (Table 3). Significant PVs included Depth, SST and X,Y (Table 4), and the parametric variable Swell was also highly significant (p <0.001). The predicted relative density of sei whales was highest over water depths of 20 to 70 m, and in grid cells with SST >7°C (S6 Fig).

**Table 3:** Performance of Generalized Additive Models (GAM) and MaxEnt models as assessed through cross-validation.

Species	RMSE	AUC
**Sei whale**	0.21	0.60
**Sei whale and large baleen whales**	0.19	0.62
**Southern right whale**	7.61e+17	0.55
**Peale’s dolphin**	0.25	0.53
**Commerson’s dolphin**	0.04	0.69

Root-mean-square error (RMSE) values are provided for the GAMs, and area under the receiver-operator curve (AUC) values are provided for MaxEnt.

**Table 4:** Summary of the 7 km resolution generalized additive model results (including effective degrees of freedom, edf) for coastal cetacean species.

Smooth PV	edf	Chi sq	P value
***Sei whale*:** Count ~ Swell + s(Depth) + s(SST, k = 6) + s(X,Y)			
Depth	3.3	14.4	0.007
SST	4.0	70.4	<0.001
X,Y	22.9	156.5	<0.001
***Sei whale and large baleen whales***: Count ~ Swell + s(Depth) + s(SST, k = 6) + s(X,Y)			
Depth	4.1	28.2	<0.001
SST	4.1	108.6	<0.001
X,Y	24.2	229.6	<0.001
***Southern right whale*:** Count ~ s(SST, k = 4) + s(ShoreDist) + s(Aspect)			
SST	2.9	45.9	<0.001
ShoreDist	1.2	4.7	0.041
Aspect	2.2	5.2	0.147
***Peale’s dolphin***: Count ~ s(Depth, k = 6) + s(ShoreDist) + s(SST, k = 6) + s(I(KelpDist^0.5))			
Depth	3.1	40.3	<0.001
ShoreDist	4.2	29.4	<0.001
SST	4.4	39.3	<0.001
KelpDist (square root transformed)	1.0	2.9	0.086
***Commerson’s dolphin***: Count ~ Swell + Sea + s(Depth) + s(SST) + s(I(Roughness^0.5))			
Depth	5.1	35.1	<0.001
SST	4.7	44.6	<0.001
Roughness (square root transformed)	1.0	2.6	0.105

See Table 1 for abbreviation definitions for the PVs.

The MaxEnt model had an AUC value of 0.60 (SD = 0.09; Table 3). SST was the most important PV and contributed 68% to the final MaxEnt model (Table 5), with the relative occurrence rate (ROR) of sei whales increasing continuously with SST (S7 Fig). ShoreDist contributed a further 14.3% to the model, with ROR predicted to remain constant with increasing distance from the coast until around 20 km from the shoreline, when a sharp increase was indicated (S7 Fig). However, the latter may represent an artefact caused by the primarily nearshore data distribution. ROR was predicted to be highest over the flattest slopes (S7 Fig).

**Table 5:** Summary of MaxEnt results for coastal cetacean species.

PV	Contribution (%)	Permutation importance
***Sei whale***		
SST	68.2	81.1
ShoreDist	14.3	5.6
Slope	7.2	3.3
Roughness	5.7	6.1
Chl-*a*	2.6	1.3
Depth	2.0	2.6
***Sei whale and large baleen whales***		
SST	61.8	70.4
Chl-*a*	13.6	5.6
ShoreDist	10.6	8.1
Slope	6.8	8.7
Roughness	3.9	3.9
Depth	3.3	3.2
***Southern right whale***		
SST	63.0	25.4
Slope	14.1	32.3
ShoreDist	13.0	22.1
Depth	9.7	19.6
Roughness	0.1	0.6
***Peale’s dolphin***		
Depth	47.3	2.4
Aspect	35.5	27.7
SST	9.7	18.9
KelpDist	2.2	12.8
Chl-*a*	2.0	12.4
Slope	1.5	7.9
ShoreDist	1.2	15.9
Roughness	0.4	2.1
***Commerson’s dolphin***		
SST	49.2	63.4
ShoreDist	14.6	4
Roughness	13.4	9.8
Depth	12.9	13.2
Slope	8.9	7.9
Chl-*a*	0.9	1.7

Predictor variables (PVs) are listed in order of percentage contribution to the model (abbreviated PV names described in Table 1).

**Fig 2:**
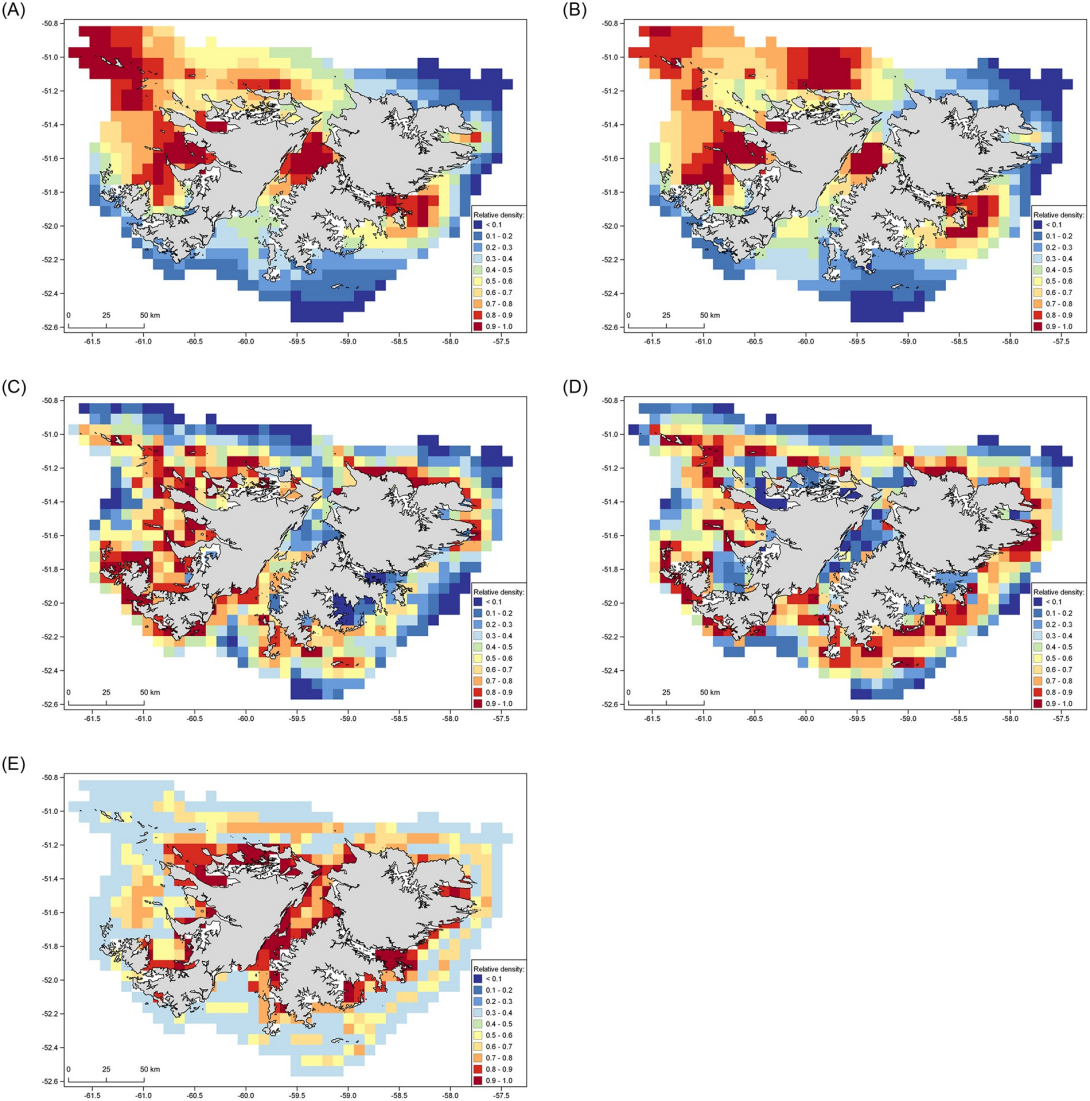
Predicted relative density of cetacean species around the Falkland Islands based on a generalized additive model at 7 km resolution. (A) sei whale. (B) Combined sei whale and large baleen whale. (C) Southern right whale. (D) Peale’s dolphin. (E) Commerson’s dolphin. The Falklands coastline shapefile was accessed open source from the Information Management System (IMS) and GIS Data Centre in Stanley, Falkland Islands (available via https://www.south-atlantic-research.org/research/data-science).

**Fig 3:**
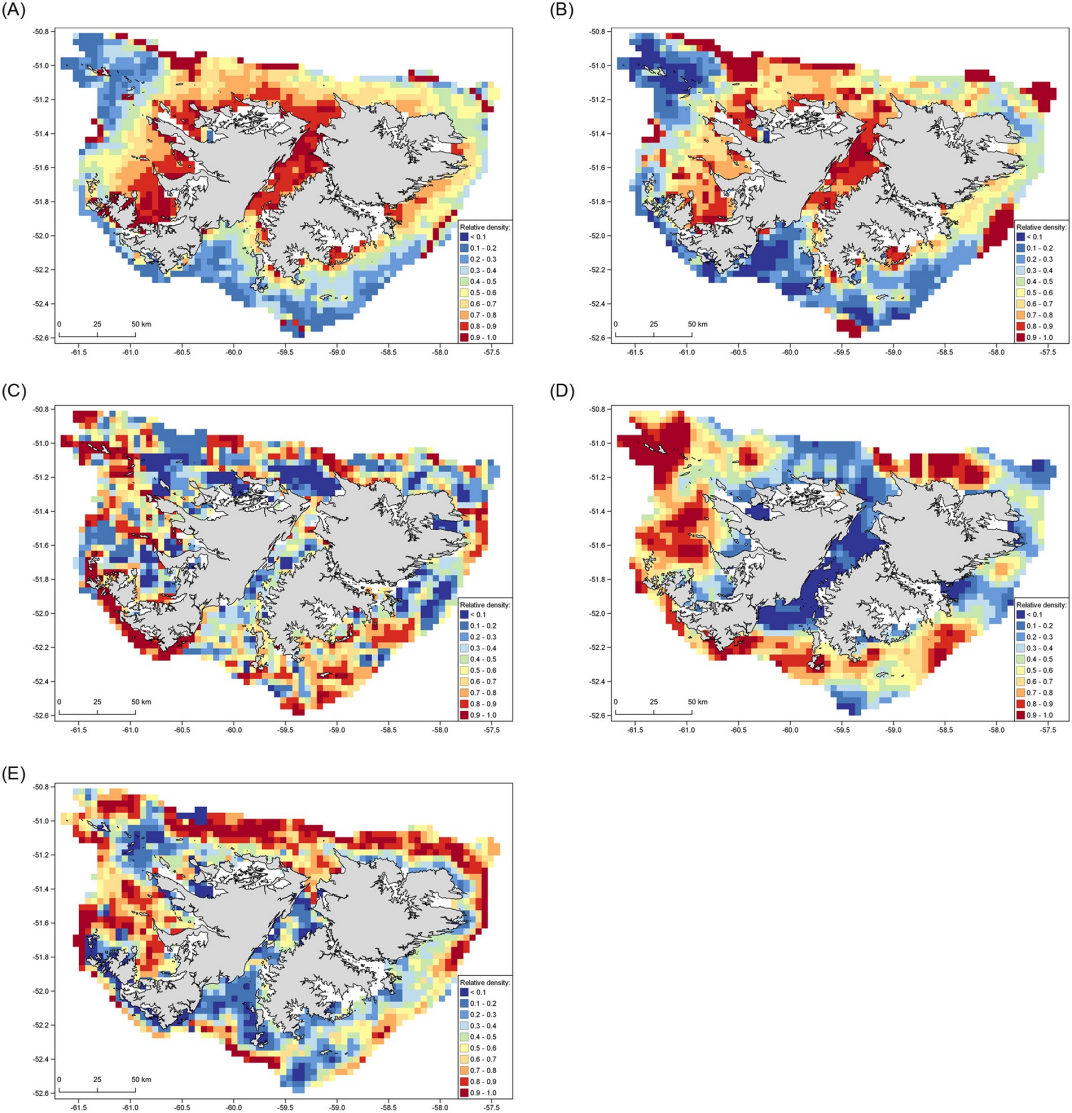
Predicted relative density of cetacean species around the Falkland Islands based on MaxEnt at 4 km resolution. (A) sei whale. (B) Combined sei whale and large baleen whale. (C) Southern right whale. (D) Peale’s dolphin. (E) Commerson’s dolphin. The Falklands coastline shapefile was accessed open source from the Information Management System (IMS) and GIS Data Centre in Stanley, Falkland Islands (available via https://www.south-atlantic-research.org/research/data-science).

There was reasonable agreement between the distribution of sei whales predicted by the GAM (Fig 2A) and MaxEnt (Fig 3A) models. Both predicted moderate to high relative sei whale densities in Berkeley Sound, north of Lively Island, within Falkland Sound, and along the west coast of the Falklands. A notable area of difference between the model predictions occurred in the north-west of the study area, where the GAM predicted high relative density around the Jason Islands while MaxEnt did not (Figs 2A and 3A). The model predictions had a reasonable overall fit with the observed sighting locations (S2 Fig), although MaxEnt better predicted whale density along the north and eastern coasts of East Falkland compared with the GAM.

### Combined sei whale and large baleen whales (SEI–BAL)

The GAM for a combined category of sei whales and unidentified large baleen whales (SEI–BAL) explained 38.9% of deviance and had a RMSE of 0.19 (Table 3). Similar to the sei whale GAM, significant PVs in the SEI–BAL model included Depth, SST and X,Y (Table 4), and the parametric variable Swell was also highly significant (p <0.001). The predicted relative density of SEI–BAL was highest over water depths of 30 to 100 m, and increased with SST reaching highest values in grid cells with SST >8°C (S8 Fig).

The MaxEnt model had an AUC value of 0.62 (SD = 0.06; Table 3). SST was the most important PV (61.8% contribution: Table 4). However, compared with the sei whale-only model, Chl-*a* was more important and contributed 13.6% to the final SEI–BAL MaxEnt model (Table 5). The ROR of SEI–BAL increased continuously with SST, but decreased with increasing Chl-*a* (S9 Fig). ShoreDist contributed an additional 10.6%, with ROR predicted to increase at distances beyond 20 km from the coast (S9 Fig).

The distributions of SEI–BAL predicted by the GAM (Fig 2B) and MaxEnt (Fig 3B) were consistent in predicting high relative whale densities in Falkland Sound, Byron Sound and Queen Charlotte Bay. They also both predicted low relative densities along the southern seaboard of the Islands. However, MaxEnt predicted more extensive areas of moderate to high relative whale density in Falkland Sound and along the north coast of the Falklands (Fig 3B), while the GAM predicted additional areas of high density around Lively Island, in King George Bay, and in the north-west region around the Jason Islands (Fig 2B). Overall, the predicted densities from the SEI–BAL models were similar to those for the models for the sei whale only dataset. The model predictions had a reasonable overall fit with the observed SEI–BAL sighting locations (S2 Fig), but similar to the sei whale model, the GAM appeared to under-predict occurrence along the northern coast of East Falkland.

### Southern right whale

The GAM for southern right whales explained 41.8% of deviance but had a high RMSE value indicating poor model performance (Table 3). Significant PVs in the right whale model included SST and ShoreDist (Table 4). While Aspect was not significant (p = 0.15), it was retained in the model as it improved the fit. The predicted relative density of southern right whales was highest in SSTs of 4–5°C, and decreased with increasing distance from the coast (S10 Fig). Predicted density was slightly higher in northerly to north-westerly aspects.

The MaxEnt model had an AUC value of 0.55 (SD = 0.32; Table 3). SST was the most important PV contributing to the model (63.0%), and Slope, ShoreDist and Depth also each contributed between 10 and 15% (Table 5). The ROR of southern right whales was predicted to be highest in cooler SSTs, with a steady decrease in occurrence as SST increased (S11 Fig). ROR also increased with slope; however, the range of slope values within the dataset was narrow at 88 to 90 degrees (S11 Fig). Right whale ROR was highest in immediate proximity to the coast, and decreased to 3 km from shore after which it stabilised (S11 Fig).

The distributions of southern right whales predicted by the two modelling approaches were inconsistent; in general, the GAM predicted higher relative densities in nearshore areas (Fig 2C), while MaxEnt predicted highest relative densities in more open waters (Fig 3C). However, both models predicted high relative densities of right whales along the south-west coast of West Falkland from the Arch Islands to New Island. The GAM predicted high relative densities along the north and north-east coasts of East Falkland, at scattered locations in the southernmost region of Falkland Sound and Sea Lion Island, and along much of the west coast from Weddell Island north to Pebble Island (Fig 2C). The MaxEnt model predicted much lower relative densities in all of those locations, instead indicating highest relative densities around the Jason Islands, north-west of the Cape Dolphin peninsular, offshore of the entrance to Berkeley Sound, and over an extensive part of the south-east coast of East Falkland (Fig 3C). Performance of the models against the observed locations of sightings was difficult to assess, since the winter right whale surveys were focussed on a spatially-limited area of NEF (S3 Fig). However, the GAM appeared to better predict occurrence in that area than MaxEnt.

### Peale’s dolphin

The GAM for Peale’s dolphin explained 17.1% of deviance and had a RMSE of 0.25 (Table 3). Significant PVs in the Peale’s dolphin model included Depth, ShoreDist and SST (Table 4). Although not significant, KelpDist was also retained as it improved the model fit. The predicted relative density of Peale’s dolphin increased steadily with Depth and was highest over water depths of 40–80 m (S12 Fig). Relative density was predicted to be highest immediately adjacent to the coast, and was then comparable across all distances from 4 to 25 km from the coast (S12 Fig). The highest predicted relative density occurred at SSTs of 6–11°C (S12 Fig), and there was a steady decrease in relative density as the distance from kelp beds increased.

The MaxEnt model had an AUC value of 0.53 (SD = 0.14; Table 3). The most important PVs were Depth and Aspect, contributing 47 and 36% respectively to the model (Table 5). ROR increased steadily with Depth across the water depth range examined in this study (1–100 m), and was highest over a south-west to north-west Aspect (S13 Fig). SST contributed a further 9.7% to the model, with ROR decreasing as SST increased and particularly in SSTs greater than 11°C (S13 Fig). KelpDist, Chl-*a*, Slope, ShoreDist and Roughness each contributed between 0.4 and 2.2% to the final model (Table 5).

The maps of predicted distribution of Peale’s dolphins differed between the two modelling approaches, with the GAM indicating highest relative densities in coastal areas whereas MaxEnt predicted highest relative densities in the more pelagic parts of the study area (Figs 2D and 3D). Both models were consistent in predicting low relative densities in the central area of Falkland Sound, north of Lively Island, Queen Charlotte Bay and Byron Sound. However, they were markedly different in their predictions for most other regions. The output from the GAM aligned better than MaxEnt with the observed distribution of Peale’s dolphin sightings (S4 Fig), although appeared to under-predict Peale’s dolphin occurrence in the waters south of Falkland Sound and within Berkeley Sound. The MaxEnt output did not correspond well with sighting locations, under-predicting occurrence around Berkeley Sound and Cape Pembroke, and along the north coast of Weddell Island.

### Commerson’s dolphin

The GAM for Commerson’s dolphins explained 41.1% of deviance and had a RMSE of 0.04 (Table 3). Significant PVs in the Commerson’s dolphin model included Depth and SST, with Roughness also being retained (Table 4). While non-significant, the parametric variables Swell and SeaState were also retained as they improved model fit. The predicted relative density of Commerson’s dolphins was highest in the shallowest water depths, with a secondary increase at 70 m depth before decreasing in deeper areas (S14 Fig). Predicted dolphin density increased with SST and also slightly with Roughness (S14 Fig).

The MaxEnt model for Commerson’s dolphins produced the highest AUC of any of the species tested, with a value of 0.69 (SD = 0.15; Table 3). The most important PVs were SST (49.2%), ShoreDist (14.6%), Roughness (13.4%) and Depth (12.9%: Table 5). The ROR of Commerson’s dolphins generally increased with SST (S15 Fig). Dolphins were predicted to occur predominantly within 10 km of the coast, declining steadily at greater distances. The ROR was highest at Roughness values of 20–30 m, and increased with Depth to around 90 m, before decreasing again in deeper water (S15 Fig).

There was poor agreement in the Commerson’s dolphin distributions predicted by the two modelling approaches. The GAM indicated high relative dolphin densities primarily extending across nearshore areas around much of the Falklands coast, particularly throughout Falkland Sound, along the north coast of West Falkland, in the innermost parts of King George and Queen Charlotte Bays, north of Bleaker and Lively Islands, and in Berkeley Sound (Fig 2E). In contrast, the highest relative densities predicted by MaxEnt were in more open waters located further from the coasts (Fig 3E), especially along the north coast of the Falkland Islands. Of the two models, the GAM had a notably better overall fit to the observed sighting locations than MaxEnt (S5 Fig), with the latter model under-predicting dolphin occurrence in most inshore areas.

## Discussion

This study used statistical modelling to further understanding of the distributions of four coastal cetacean species around the Falkland Islands. The primary objective was to apply a statistically robust method of interpolating the field data and using it for predictive distribution mapping [4], in order to generate information on the potential occurrence of cetaceans in areas that had not yet been surveyed. Gaining insights into the important PVs that might drive those distributions was a secondary aim.

### Model performance

For all of the cetacean species modelled in the Falklands, a 7 km resolution GAM produced better model fit than a finer resolution GAM (4 km resolution), indicating that although finer resolutions may seem advantageous from a management perspective, they can create a false impression of precision. This is especially true for dynamic marine ecosystems, where spatial variability can occur at scales of up to thousands of kilometres [10]. A 4 km resolution was the lowest spatial-scale considered in the GAM or MaxEnt models, because it represented the best available resolution of the SST and Chl-*a* satellite imagery. Finer-scales also potentially introduce issues with the correlation between the locations of effort legs and associated sightings, with more sightings falling into adjacent cells to where the associated effort occurred as the grid cell resolution decreases. The selection of the largest grid cell size considered in our models was limited by the complexity of the coastal study site, and the desire (for informing management) to be able to predict species occurrence inside topographic features such as inlets and bays which may not be captured at larger scales.

The choice of model type and the resolution of PVs are key technical issues affecting model applicability [10, 22]. Moreover, validation of model outputs remains problematic, especially where logistic and economic constraints inhibit ground-truthing in the field. We addressed this through the use of cross-validation, which was automatically incorporated into the MaxEnt model process (i.e. AUC), but which we additionally applied to assess GAM performance (i.e. RMSE). The species for which the models performed best (i.e. lowest RMSE and highest AUC values) were the same for both GAM and MaxEnt, comprising Commerson’s dolphin, combined SEI–BAL, and sei whales. Concurrently, the GAM results explained between 34.7 and 41.1% of the total deviance for those species, which is similar to, or better than, the values achieved for cetacean species in many other studies [e.g. 8, 35, 36]. The MaxEnt models for those three species had moderate discriminatory power with AUC values of 0.60 to 0.69, although those values are lower than those achieved for MaxEnt modelling of cetacean species in some other studies [e.g. 6, 16, 37]. The validation techniques indicated a slightly improved performance of both GAMs and MaxEnt for combined SEI–BAL compared with sei whales only, likely reflecting the larger sample size available for the former.

Although the Peale’s dolphin GAM performed well (RMSE = 0.25) it explained only 17.1% of the total deviance, suggesting that other factors may be influencing the occurrence of that species. Similarly, the MaxEnt model for Peale’s dolphins had the lowest AUC of any of the species considered, performing only slightly better than random. The poorest performing models were those for the southern right whale. While the GAM explained 41.8% of deviance for southern right whales which was the highest for any of the species, the large RMSE value produced by the cross-validation indicated that the model had poor predictive performance. The MaxEnt AUC value of 0.55 also indicated a performance only slightly better than random. Potential explanations for the lower performance of Peale’s dolphin and southern right whale models are considered in the species sections below.

Differences were also apparent between the two modelling approaches with regard to the predicted species distributions and the relative significance of the PVs for each species. For sei whales and combined SEI–BAL, the GAM and MaxEnt outputs had satisfactory agreement. Given the poor performance of the southern right whale models, comparisons between the model results for that species are unlikely to be meaningful. With respect to the two dolphin species, the MaxEnt model generally predicted a higher ROR in offshore parts of the study area while the GAMs predicted highest relative density in coastal parts of the study area; the GAM had better overall fit with the distribution of sightings for both species. Two relevant technical differences in the modelling methods may have contributed to such differences in the outputs. Firstly, the way in which the PVs are incorporated into the models differs between the GAM and MaxEnt approaches. We used a step-wise additive approach in the GAM construction, effectively testing every combination of PVs and retaining only those that contributed to model fit [27], resulting in only a small number of PVs being retained in the final models. In contrast, MaxEnt retained all PVs in each model, but weighted their contributions differently. Secondly, the method by which survey effort is accounted for differs between the models. For the GAMs, effort was included as an offset (including for grid cells were no animals were recorded), and therefore zero-inflation may have affected GAM performance for species with fewer sightings. In contrast, a raster of overall effort distribution was incorporated into MaxEnt (i.e. without accounting for temporal variation in effort or environmental conditions), and the use of positive presence records meant that the model was not affected by zero-inflation of data. Consequently, while zero-inflation may have degraded the performance of the GAMs, the mechanism for accounting for effort bias is weaker in Maxent and less impacted by zero-inflation. The higher numbers of sightings of sei whales and combined SEI–BAL compared with Peale’s and Commerson’s dolphins, may therefore have produced better-performing GAMs and less variation between the results of the two modelling approaches.

Several additional factors may have influenced the performance of both model approaches during this study. Cetaceans are highly mobile marine predators, exhibiting a high degree of spatial variability over a fine temporal scale relative to the scale of the seasonally variable PVs used. Monthly aggregate values were used for SST and Chl-*a*, since atmospheric conditions over the Falkland Islands resulted in incomplete remote sensing data at finer temporal scales. The predictive power of those PVs may potentially have increased for each species if a finer-scale dataset had been available. Some studies have collected in situ data on PVs to address that issue [e.g. 8], although others have concluded that the use of satellite datasets had predictive ability that met or exceeded that of in situ data [11]. Additionally, the relationship between some species and the PVs may be present, but with temporal or spatial lags between physical processes and biological responses [10, 16, 37]. For example, a North Atlantic study found that May SST was consistently one of the strongest predictors of sei whale distribution recorded during survey effort two months later in July, suggesting that the May temperatures may influence productivity and prey density later in the year [12].

SST was the PV that contributed most significantly to all of the GAM and MaxEnt models, with the predicted relative densities of sei whales, combined SEI–BAL, and Commerson’s dolphins all increasing with SST, while southern right whales exhibited the opposite trend. The predicted relative density of Peale’s dolphins did not exhibit clear trends with SST; rather, that species appeared to prefer the mid-range of water temperatures occurring within the study area. Although only a relatively narrow range of water depths was surveyed and modelled, Depth was also a consistently important PV in most models. There may be other important explanatory variables influencing the distribution of cetaceans in this study that were not incorporated as PVs, in particular direct measures of prey distribution and abundance. Several studies have demonstrated that due to the mobility and spatial variability of cetacean prey species, it may be preferable to use environmental variables as proxies of their occurrence [13, 38]. However, that relies upon having robust information regarding the PVs that may be governing those prey, and of the extent to which cetacean species specialise on particular prey species rather than switching between available prey [9]. Little is known of the diets of coastal cetacean species around the Falkland Islands, and where evidence of prey species does exist then no data are currently available on the extent of prey specialism or on the factors influencing the spatio-temporal distribution of those prey around the Falklands.

The power of models to predict the spatial probability of cetacean occurrence also depends on behavioural considerations. The visual dataset collected in the Falklands was necessarily restricted to daylight hours. However, if species exhibit differing habitat preferences, associated with different behavioural activities, during the day compared to night, then that may limit the predictive power of certain PVs and the overall models.

### Sei whales

The modelled results for sei whale and for SEI–BAL were broadly similar with regard to important PVs and overall predicted distributions, which is consistent with the supposition that most, if not all, unidentified baleen whales comprised sei whales.

Sei whales are a highly mobile species, with photo-identification and suction-cup tagging data in the Falklands indicating that their movements vary individually such that some individuals remain in the same areas for several weeks while others move tens of kilometres overnight [Falklands Conservation, unpublished data]. Therefore, sightings will be recorded both in key foraging habitats and in less optimal areas through which the animals are simply transiting, potentially affecting the predictive power of the model. Nevertheless, the final GAM models for sei whales and SEI–BAL explained 35–39% of the total deviance, which is similar to other studies modelling balaenopterids on their feeding grounds, for example fin whales in the Gulf of St. Lawrence (23.7%: [13]), Antarctic minke whales in the Southern Ocean (25.3%: [39]), and sei whales in the North Pacific (44.9%: [40]).

Sei whales use the coastal waters around the Falkland Islands as a seasonal feeding ground, preying on shoaling species including the crustacean squat lobster (*Munida gregaria*) and the amphipod *Themisto gaudichaudii* [21]. The occurrence of sei whales on feeding grounds in the North Atlantic and North Pacific has been associated with topographic features such as depth [12], and with meso-scale (>100 km) oceanic features including fronts, eddies, gyres, and upwelling regions where ocean currents interact with seabed topography and are presumed to concentrate prey and increase foraging efficiency [e.g. 40–43). However, it is clear that sei whales in the Falkland Islands are feeding in very different, inner shelf, habitats compared to the oceanic habitats described in the Northern Hemisphere. The importance of topographic PVs including Depth in the GAMs, and ShoreDist and Slope in the MaxEnt models, are all indicative of an occurrence in rather shallow, flat, and coastal habitat. Since the GAM predictions were generated using satellite PV data from March, the importance of SST in predicting sei whale and SEI–BAL relative density relates specifically to the month of their known peak seasonal occurrence on the Falklands feeding ground [21]. This suggests that SST is an important PV determining the fine-scale distribution of foraging sei whales. It may potentially reflect greater prey availability associated with warmer water temperatures, for example the occurrence of *Munida* swarms higher in the water column during summer compared with winter [44]. Chl-*a* was also an important PV for SEI–BAL in MaxEnt, which is consistent with other studies indicating that baleen whale distribution in feeding areas is linked to primary productivity, presumably since it drives the occurrence of prey species [12, 36, 37, 45].

In the oceanic North Pacific, sei whale foraging dives were shallower at night in response to vertical migration of their prey species towards the surface, but they continued to undertake foraging dives throughout the daytime [46]. Consequently, if sei whales are assumed to maintain foraging dives throughout the day, then the distributions predicted from visual survey data collected during daylight hours around the Falkland Islands are likely to broadly reflect those of their prey species. Unfortunately, no studies have examined the spatio-temporal abundance of *Munida* or *Themisto* in the Falklands. Existing information on *Munida* from elsewhere on the Patagonian Shelf suggests that swarms remain in the same areas year-round but are larger and occur deeper in the water column during winter [44], and that diel vertical migration by various *Munida* life stages is less apparent than vertical movements related to the tidal cycle [47]. Although costly, the collection of targeted data on the abundance, distribution and behaviour of prey species, and particularly the collection of concurrent real-time data on prey biomass during whale surveys, may help to better explain sei whale occurrence in the Falklands. However, inclusion of prey biomass data does not always result in better predictive performance for more generalist baleen whale species [e.g. 13].

### Southern right whales

Both models, particularly the GAM, performed poorly for southern right whales, and were not considered to have generated meaningful results. There are several potential explanations for this outcome. Firstly, the sightings of southern right whales recorded during the boat surveys contrasted with the other species in: (1) being limited to the austral winter between mid-May and August; and (2) occurring only during surveys in the NEF study area. Those points are related, since surveys in WF and FS occurred predominantly during the summer and autumn. Consequently, extrapolation of southern right whale predictions from data collected at one small site to the entirety of the Falkland Islands introduces extra uncertainties.

Secondly, it is plausible that southern right whales genuinely exhibit fewer habitat preferences in the study area, since their occurrence along the Falklands coast in winter appears to be related to socialising, courtship and migratory behaviour [24], rather than being driven by foraging as for the other cetacean species. Their seasonal migration from higher-latitude feeding areas to their winter breeding grounds along the coasts of Argentina and Brazil brings some right whales close to the coast of the Falklands, and such long-range transits across a wide variety of water depths and temperatures clearly indicate the tolerance of migrating whales to a variety of habitats. Although wintering grounds elsewhere share consistencies in being shallow in depth, located close to shore, over sandy seabeds, and sheltered from prevailing swell and wind [48], differences in habitat preferences are apparent between whales depending on their reproductive status. Cow-calf pairs have stronger preferences and favour the most sheltered habitats, while non-calf groups exhibit a wider tolerance for habitat features and consequently may have poorer model fit [48]. During winter in the Falklands, southern right whale sightings comprise socialising adults and juveniles, with no confirmed records to date of mother-calf pairs [24]. Consequently, it might be expected that this component of the population may have the broadest habitat requirements. Furthermore, the winter occurrence of right whales on their Argentinean breeding areas appears to be most strongly determined by the presence of other whales rather than by specific environmental parameters, with loose herds moving together back and forth along sections of coast tens of kilometres in extent [49].

Thirdly, it is possible that the winter distribution of southern right whales in the NEF may be influenced by factors other than those considered in this study. Additional PVs such as wave exposure and substrate type could be incorporated into future models; however, the collection of winter survey data along other coastlines that experience different prevailing conditions would be fundamental to that approach. Winter surveys in other regions around the Falkland Islands are also needed to increase the spatial extent of the dataset, which may improve future model results.

### Dolphins

The modelling of Peale’s and Commerson’s dolphins supported a broadly sympatric distribution across the area of inner Falklands shelf considered in this study, with the predicted relative densities of both species showing overlap. Indeed, several sightings of mixed-species schools were recorded during the boat surveys. However, there were notable differences between the GAM and MaxEnt predicted distributions for both dolphin species, and the two models for Peale’s dolphins had the lowest predictive power of all of the cetacean species models considered in this study. In general, the GAM outputs were considered a better fit to the sighting locations for both dolphin species. The GAM distributions, and to a lesser extent those of MaxEnt, predicted higher relative densities of Commerson’s dolphins in nearshore, semi-enclosed, waters compared with Peale’s dolphins, and are indicative of some habitat partitioning between the dolphin species. Consequently, Commerson’s dolphin relative densities were higher within FS and in the bays along WF, while Peale’s dolphin densities were generally higher, and located slightly further from shore, in exposed, open habitats. This result is comparable with studies of these species elsewhere [e.g. 50], and with other sympatric dolphin species in the *Lagenorhynchus* and *Cephalorhynchus* genera [51]. For example, similar habitat partitioning has been described between another South American *Cephalorhynchus* species, the Chilean dolphin (*C*. *eutropia*), and Peale’s dolphins in the Chiloé region of Chile, where Peale’s dolphins tended to occupy a wider range of habitats and were more prevalent along open or exposed coastlines [8]. It is presumed that differences in prey species are the drivers of such partitioning, but little information is available on the diet of Peale’s or Commerson’s dolphins in the Falklands. It is plausible that either (or both) species may exhibit diurnal inshore-offshore movements related to feeding, as has been reported for some members of the *Cephalorhynchus* genus [51]. Indeed, such movements are suspected in the Falklands for Peale’s dolphins based on anecdotal observations of dolphins resting and socialising in nearshore areas during the day, and moving offshore away from those areas during early evening. Any such diurnal differences in habitat use could be expected to limit the predictive power of the models.

Some studies have found associations between Peale’s dolphins and kelp beds [52]. Our study found no significant correlation between either dolphin species and distance to kelp beds, although KelpDist did improve the fit of the Peale’s dolphin GAM and contributed 2.2% to the MaxEnt model. Both Peale’s and Commerson’s dolphins do use kelp beds in the Falkland Islands, but both species also occur in open shelf habitat at considerable distance from kelp beds. Consequently, the lack of significant association with kelp in our study may be a consequence of the greater amount of boat-based survey effort in open, more pelagic habitats (i.e. up to 25 km from shore) compared with the very nearshore distribution of survey effort (e.g. dolphins tracked from shore-based observation sites) in some other studies [52]. Additionally, if the strength of relationships between dolphin species and kelp beds varies temporally (for example, if those associations are particularly strong in summer), any such relationships may potentially be diluted by the wide seasonal coverage of our dataset.

The most important PVs for predicting the occurrence of Commerson’s dolphins in the Falklands (i.e. SST, ShoreDist and Depth) were also important factors determining the occurrence of Chilean dolphins (*C*. *eutropia*) in Chile [8], and are consistent with the coastal habitats favoured by all species in that genus. Also consistent between the Falklands and Chile, was the higher amount of variance explained for *Cephalorhynchus* species compared to Peale’s dolphins in the models [8], again supporting previous indications that Peale’s dolphins have generally broader habitat preferences across their range [50].

The lack of available satellite imagery for large portions of the innermost inlets and bays, limits some of the interpretation of predicted dolphin occurrence in those areas. In many cases those are areas known to be favoured by Commerson’s dolphins, and the lack of available satellite data in many of the grid cells where Commerson’s dolphin sightings were recorded is likely to explain the under-predicted occurrence of that species in some nearshore areas.

### Conservation implications

This study has modelled a survey dataset collected around the Falkland Islands, and used it to predict the occurrence of four cetacean species across inner shelf habitat including areas where existing survey coverage was either scant or absent. The Falkland Islands are a relatively remote and sparsely-populated archipelago, with a complex coastline, and annual mean wind speeds of 28 km/hr. Consequently, conducting monitoring surveys for cetaceans around the Islands is logistically challenging and costly. Predictive habitat modelling, while requiring cautionary interpretation, offers one method to scale-up from local boat-based survey data to predict distribution across a wider area of shelf waters [5]. This information can inform the prioritisation of resources for future survey work (e.g. where best to target focal surveys).

The modelling results may be useful in identifying areas where species occurrence and anthropogenic activities are most likely to overlap, which is especially relevant to marine spatial planning [16, 35]. Human activities in shelf waters around the Falklands include shipping (such as cruise vessels, tankers and supply ships), fishing, operations related to the oil and gas industry, and the potential development of an aquaculture industry. It has been inferred by some studies that anthropogenic impacts on sei whales may be lower than for other baleen whales due to their predominantly oceanic global distribution [e.g. 53]. However, around southern South America such as Chile and the Falkland Islands, sei whales frequently inhabit neritic and coastal habitat, increasing their overlap with human activities and potential exposure to threats such as vessel strikes and entanglements [e.g. 54].

In many areas, predictive habitat modelling has also been used to aid development of spatially-explicit management areas [e.g. 5, 8]. This was the primary driver of the current study, in order to use the model outputs to inform the delineation of a potential KBA for sei whales in the Falkland Islands. The sei whale and SEI–BAL models of predicted relative density support a wide distribution across the inner shelf waters around the Falklands, and consequently it is recommended that any KBA should be sufficiently large to incorporate those areas of suitable habitat. Moreover, in recognition of the dynamic nature of marine habitats, the high mobility of sei whales, and the proven connectivity between different areas of the Falkland Islands shown by photo-identification recaptures of individual sei whales [21], it is likely that a single island-wide KBA may be more ecologically-relevant to conserving sei whales than multiple smaller KBAs.

## Supporting information

S1 Fig Relationship between grid cell size and the proportion of sightings in which the observer and the sighting were located within the same grid cell after sighting positions had been recalculated based on distance and angle from the platform at the time of initial detection. The proportion of within-cell sightings increases with grid cell resolution, although some sightings may always be located in different grid cells from the observer due to instances where the initial detection occurs close to a cell boundary. The most appropriate grid cell size was selected based on the overall aim to carry out the analysis at the finest-scale resolution possible, while also avoiding too many sightings occurring in adjacent grid cells (i.e. ≤6 km resolution).
(PNG)

S2 Fig On-effort sightings of sei whales and unidentified large baleen whales, 2017–2019. Boat-based survey effort (all weather) is shown in red. Associated sighting locations are recalculated based on angle and distance. The Falklands coastline shapefile was accessed open source from the Information Management System (IMS) and GIS Data Centre in Stanley, Falkland Islands (available via https://www.south-atlantic-research.org/research/data-science).
(TIFF)

S3 Fig On-effort sightings of southern right whales, 2017–2019. Boat-based survey effort (all weather) for May to August is shown in red. Associated sighting locations are recalculated based on angle and distance. The Falklands coastline shapefile was accessed open source from the Information Management System (IMS) and GIS Data Centre in Stanley, Falkland Islands (available via https://www.south-atlantic-research.org/research/data-science).
(TIFF)

S4 Fig On-effort sightings of Peale’s dolphins, 2017–2019. Boat-based survey effort (all weather) is shown in red. Associated sighting locations are recalculated based on angle and distance. The Falklands coastline shapefile was accessed open source from the Information Management System (IMS) and GIS Data Centre in Stanley, Falkland Islands (available via https://www.south-atlantic-research.org/research/data-science).
(TIFF)

S5 Fig On-effort sightings of Commerson’s dolphins, 2017–2019. Boat-based survey effort (all weather) is shown in red. Associated sighting locations are recalculated based on angle and distance. The Falklands coastline shapefile was accessed open source from the Information Management System (IMS) and GIS Data Centre in Stanley, Falkland Islands (available via https://www.south-atlantic-research.org/research/data-science).
(TIFF)

S6 Fig Smoothing curves of the number of sei whales recorded against the predictor variables retained by GAM. Dotted lines represent 95% confidence intervals. Degrees of freedom are show in parentheses on the y-axis label. The vertical lines above the x-axis show positions of the measured data points.
(JPG)

S7 Fig MaxEnt response curves demonstrating how each significant PV in the sei whale model affected the MaxEnt prediction. The curves show how the predicted relative occurrence rate changes as each PV is varied, keeping all other PVs at their average sample value. The curves represent the mean response of 20 replicate MaxEnt runs (red) and the mean +/- one standard deviation (blue, two shades for categorical variables).
(TIF)

S8 Fig Smoothing curves of the number of combined sei and large baleen whales recorded against the predictor variables retained by GAM. Dotted lines represent 95% confidence intervals. Degrees of freedom are show in parentheses on the y-axis label. The vertical lines above the x-axis show positions of the measured data points.
(JPG)

S9 Fig MaxEnt response curves demonstrating how each significant PV in the combined sei and large baleen whale model affected the MaxEnt prediction. The curves show how the predicted relative occurrence rate changes as each PV is varied, keeping all other PVs at their average sample value. The curves represent the mean response of 20 replicate MaxEnt runs (red) and the mean +/- one standard deviation (blue, two shades for categorical variables).
(TIF)

S10 Fig Smoothing curves of the number of southern right whales recorded against the predictor variables retained by GAM. Dotted lines represent 95% confidence intervals. Degrees of freedom are show in parentheses on the y-axis label. The vertical lines above the x-axis show positions of the measured data points.
(JPG)

S11 Fig MaxEnt response curves demonstrating how each significant PV in the southern right whale model affected the MaxEnt prediction. The curves show how the predicted relative occurrence rate changes as each PV is varied, keeping all other PVs at their average sample value. The curves represent the mean response of 20 replicate MaxEnt runs (red) and the mean +/- one standard deviation (blue, two shades for categorical variables).
(TIF)

S12 Fig Smoothing curves of the number of Peale’s dolphins recorded against the predictor variables retained by GAM. Dotted lines represent 95% confidence intervals. Degrees of freedom are show in parentheses on the y-axis label. The vertical lines above the x-axis show positions of the measured data points.
(TIF)

S13 Fig MaxEnt response curves demonstrating how each significant PV in the Peale’s dolphin model affected the MaxEnt prediction. The curves show how the predicted relative occurrence rate changes as each PV is varied, keeping all other PVs at their average sample value. The curves represent the mean response of 20 replicate MaxEnt runs (red) and the mean +/- one standard deviation (blue, two shades for categorical variables).
(TIF)

S14 Fig Smoothing curves of the number of Commerson’s dolphins recorded against the predictor variables retained by GAM. Dotted lines represent 95% confidence intervals. Degrees of freedom are show in parentheses on the y-axis label. The vertical lines above the x-axis show positions of the measured data points.
(JPG)

S15 Fig MaxEnt response curves demonstrating how each significant PV in the Commerson’s dolphin model affected the MaxEnt prediction. The curves show how the predicted relative occurrence rate changes as each PV is varied, keeping all other PVs at their average sample value. The curves represent the mean response of 20 replicate MaxEnt runs (red) and the mean +/- one standard deviation (blue, two shades for categorical variables).
(TIF)

S1 File Dataset used for analysis. GIS shapefile containing effort and sighting points.
(ZIP)
