# Supplementary figures and images for: Predicting suitable coastal habitat for sei whales, southern right whales and dolphins around the Falkland Islands
Source: PLoS One. 2020 Dec 23;15(12):e0244068. doi: 10.1371/journal.pone.0244068 (PMC7757899; doi:10.1371/journal.pone.0244068)

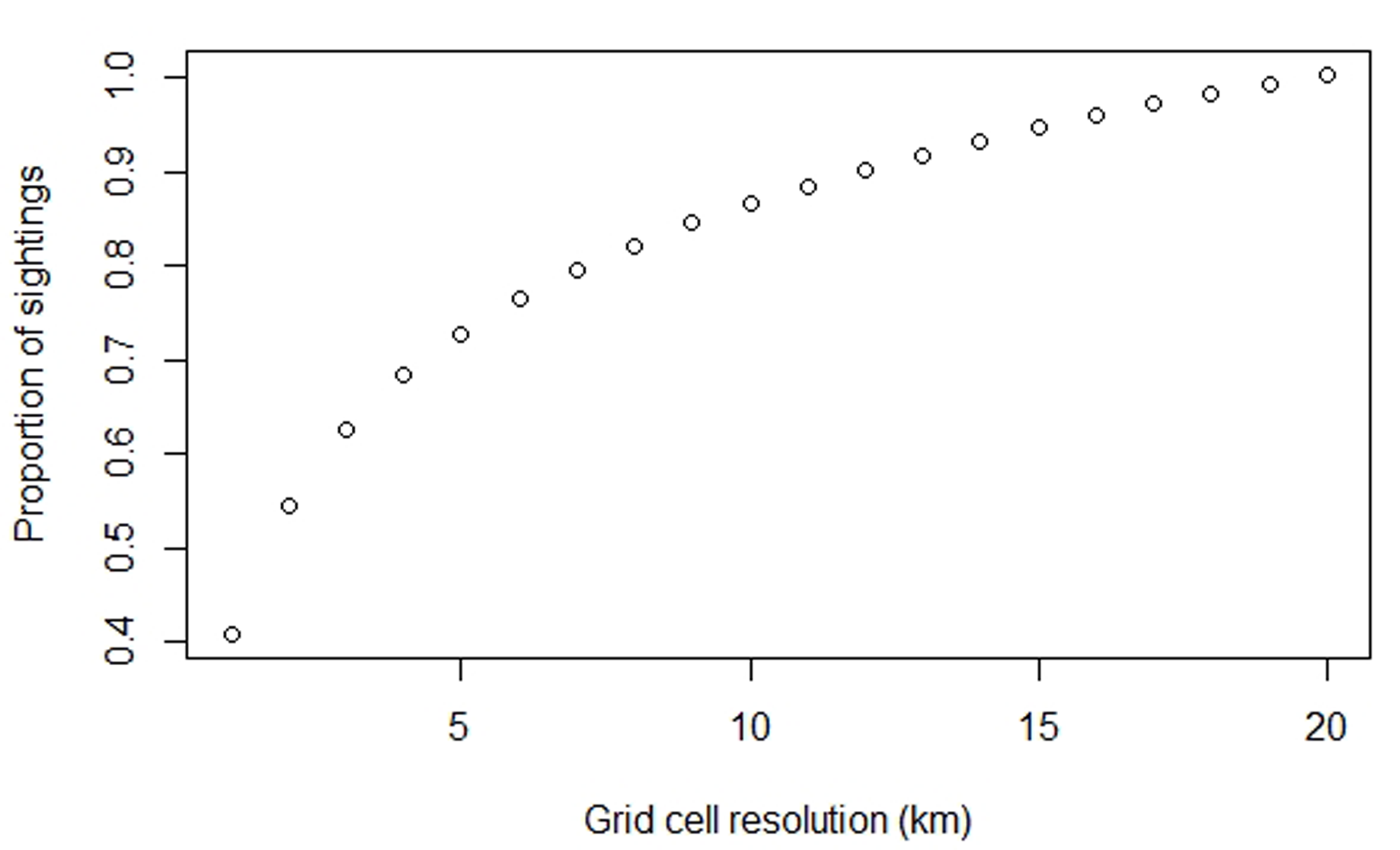

Supplement: S1 Fig — The proportion of within-cell sightings increases with grid cell resolution, although some sightings may always be located in different grid cells from the observer due to instances where the initial detection occurs close to a cell boundary. The most appropriate grid cell size was selected based on the overall aim to carry out the analysis at the finest-scale resolution possible, while also avoiding too many sightings occurring in adjacent grid cells (i.e. ≤6 km resolution). (PNG) [file pone.0244068.s001.png]

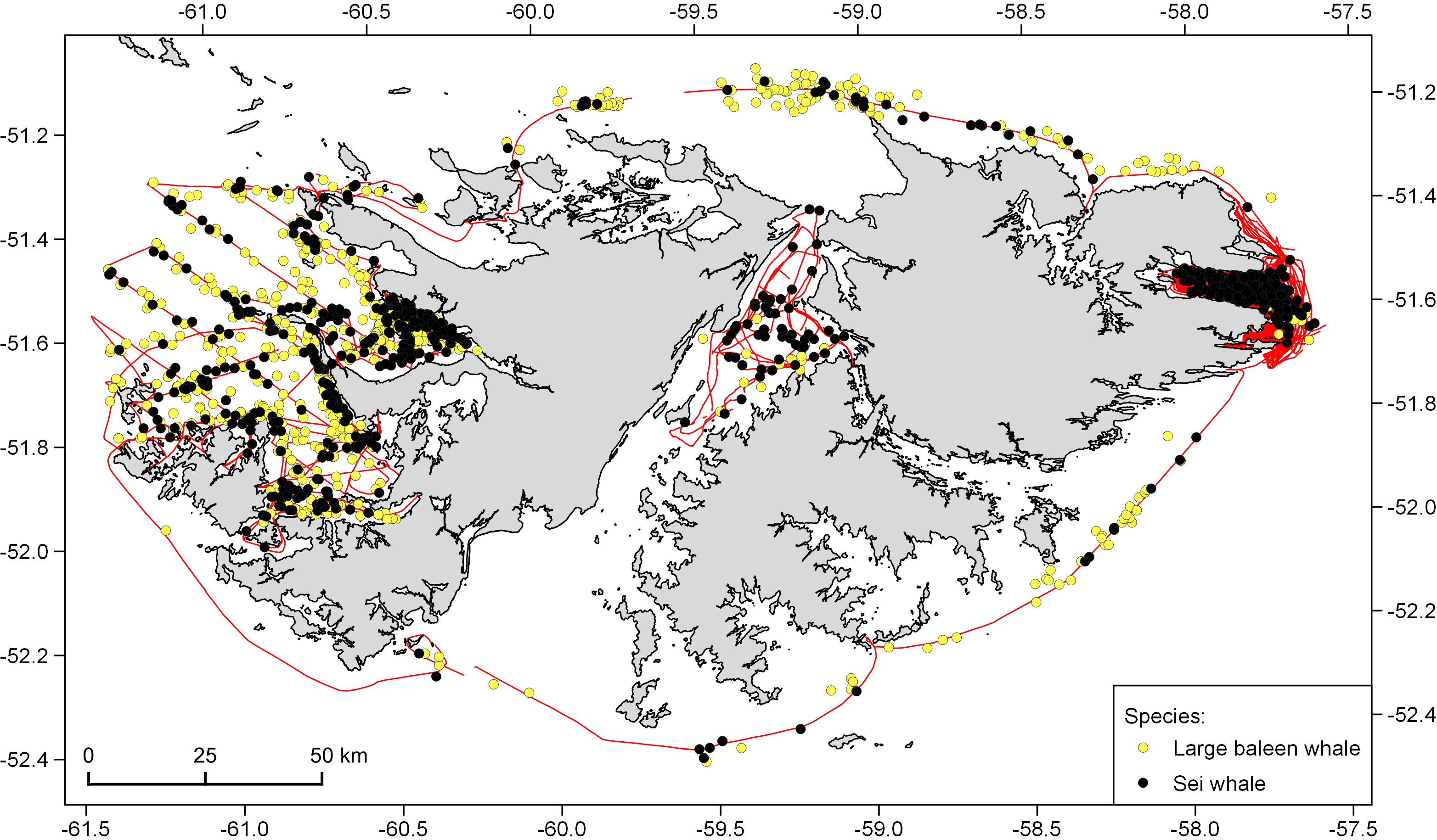

Supplement: S2 Fig — Boat-based survey effort (all weather) is shown in red. Associated sighting locations are recalculated based on angle and distance. The Falklands coastline shapefile was accessed open source from the Information Management System (IMS) and GIS Data Centre in Stanley, Falkland Islands (available via https://www.south-atlantic-research.org/research/data-science). (TIFF) [file pone.0244068.s002.tiff]

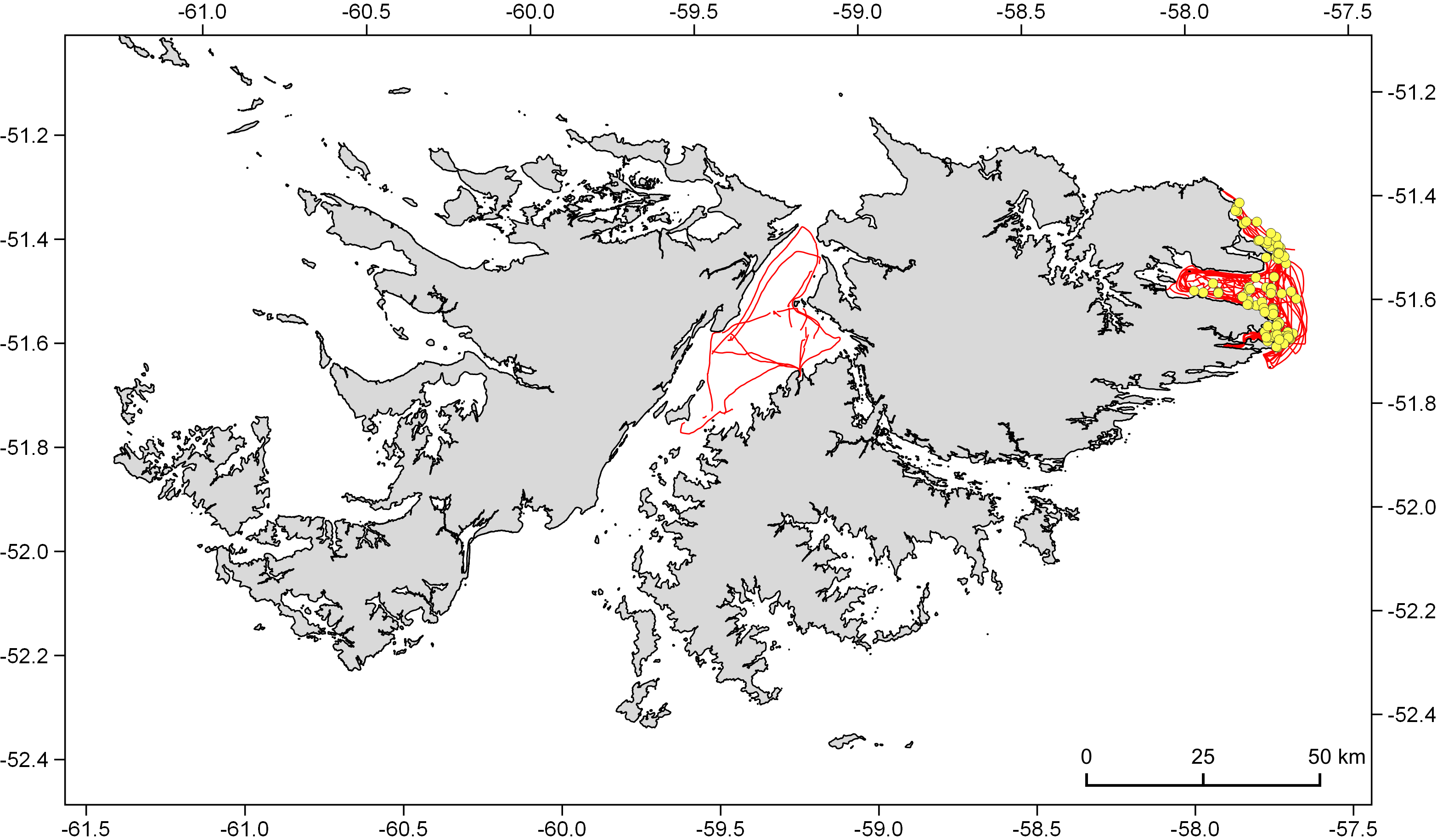

Supplement: S3 Fig — Boat-based survey effort (all weather) for May to August is shown in red. Associated sighting locations are recalculated based on angle and distance. The Falklands coastline shapefile was accessed open source from the Information Management System (IMS) and GIS Data Centre in Stanley, Falkland Islands (available via https://www.south-atlantic-research.org/research/data-science). (TIFF) [file pone.0244068.s003.tiff]

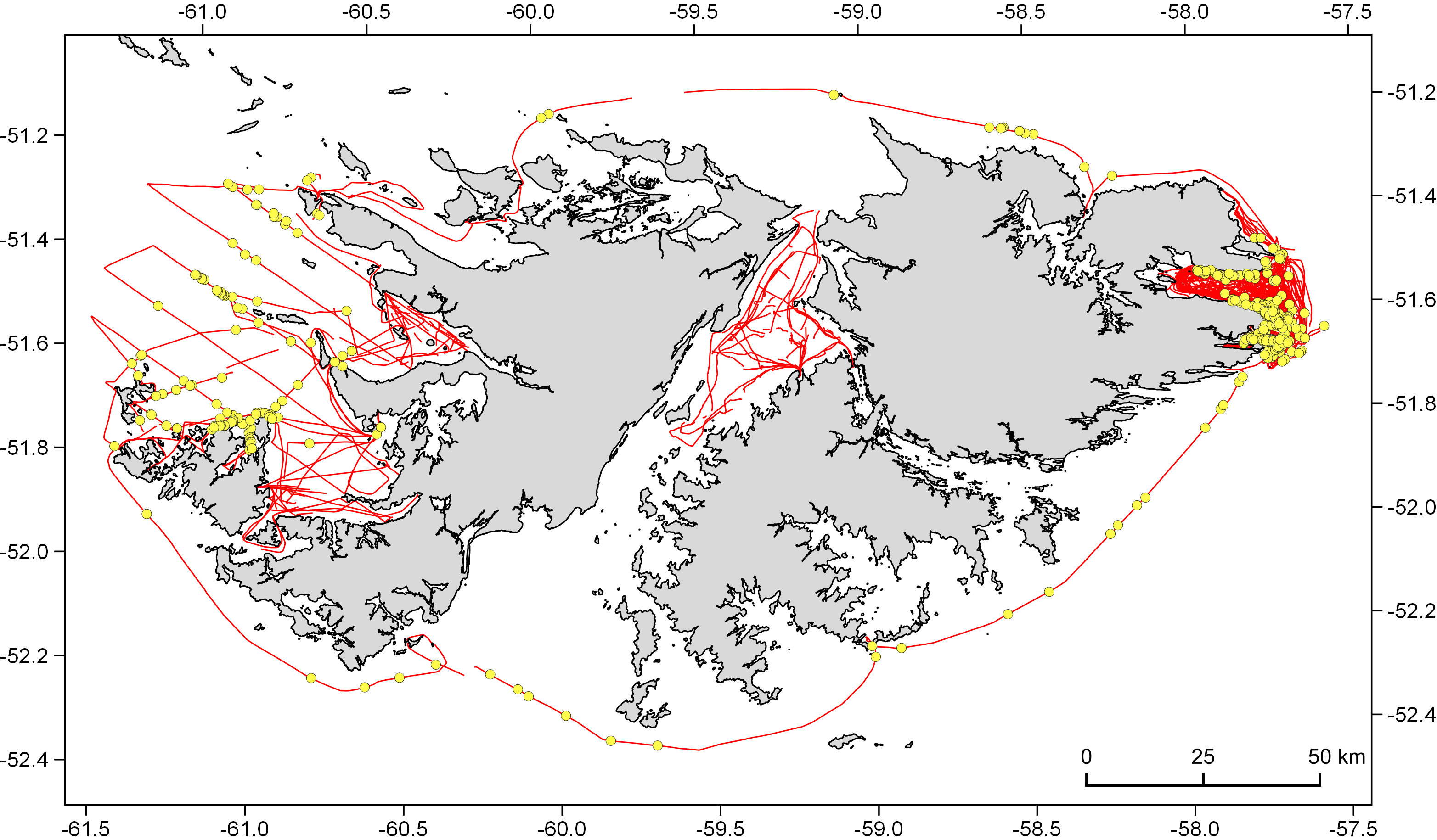

Supplement: S4 Fig — Boat-based survey effort (all weather) is shown in red. Associated sighting locations are recalculated based on angle and distance. The Falklands coastline shapefile was accessed open source from the Information Management System (IMS) and GIS Data Centre in Stanley, Falkland Islands (available via https://www.south-atlantic-research.org/research/data-science). (TIFF) [file pone.0244068.s004.tiff]

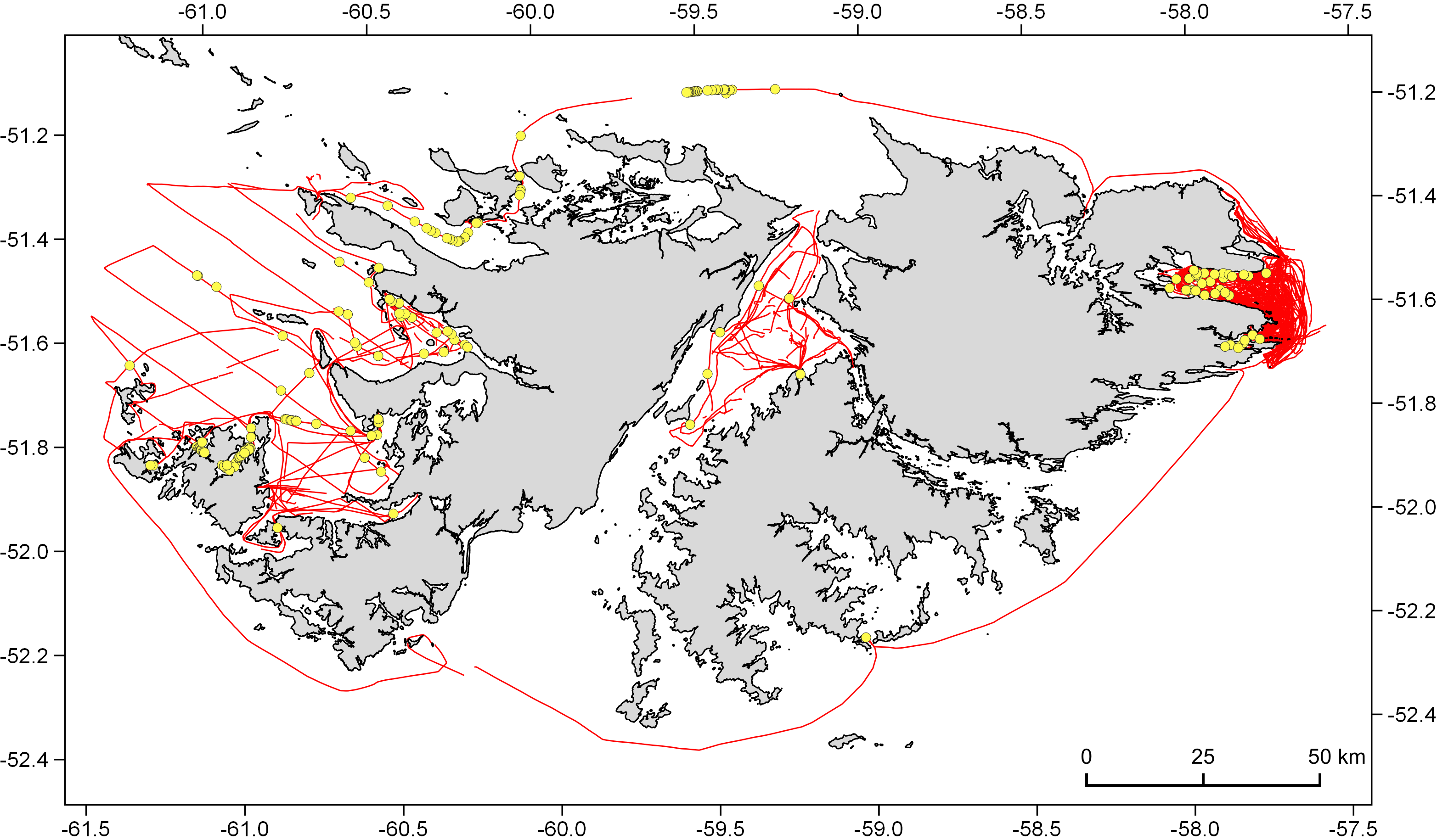

Supplement: S5 Fig — Boat-based survey effort (all weather) is shown in red. Associated sighting locations are recalculated based on angle and distance. The Falklands coastline shapefile was accessed open source from the Information Management System (IMS) and GIS Data Centre in Stanley, Falkland Islands (available via https://www.south-atlantic-research.org/research/data-science). (TIFF) [file pone.0244068.s005.tiff]

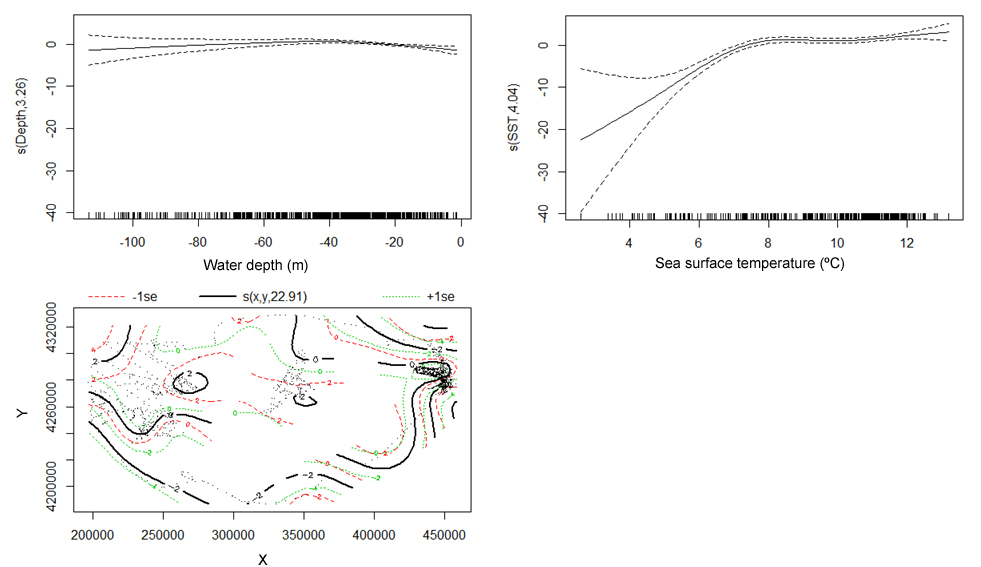

Supplement: S6 Fig — Dotted lines represent 95% confidence intervals. Degrees of freedom are show in parentheses on the y-axis label. The vertical lines above the x-axis show positions of the measured data points. (JPG) [file pone.0244068.s006.jpg]

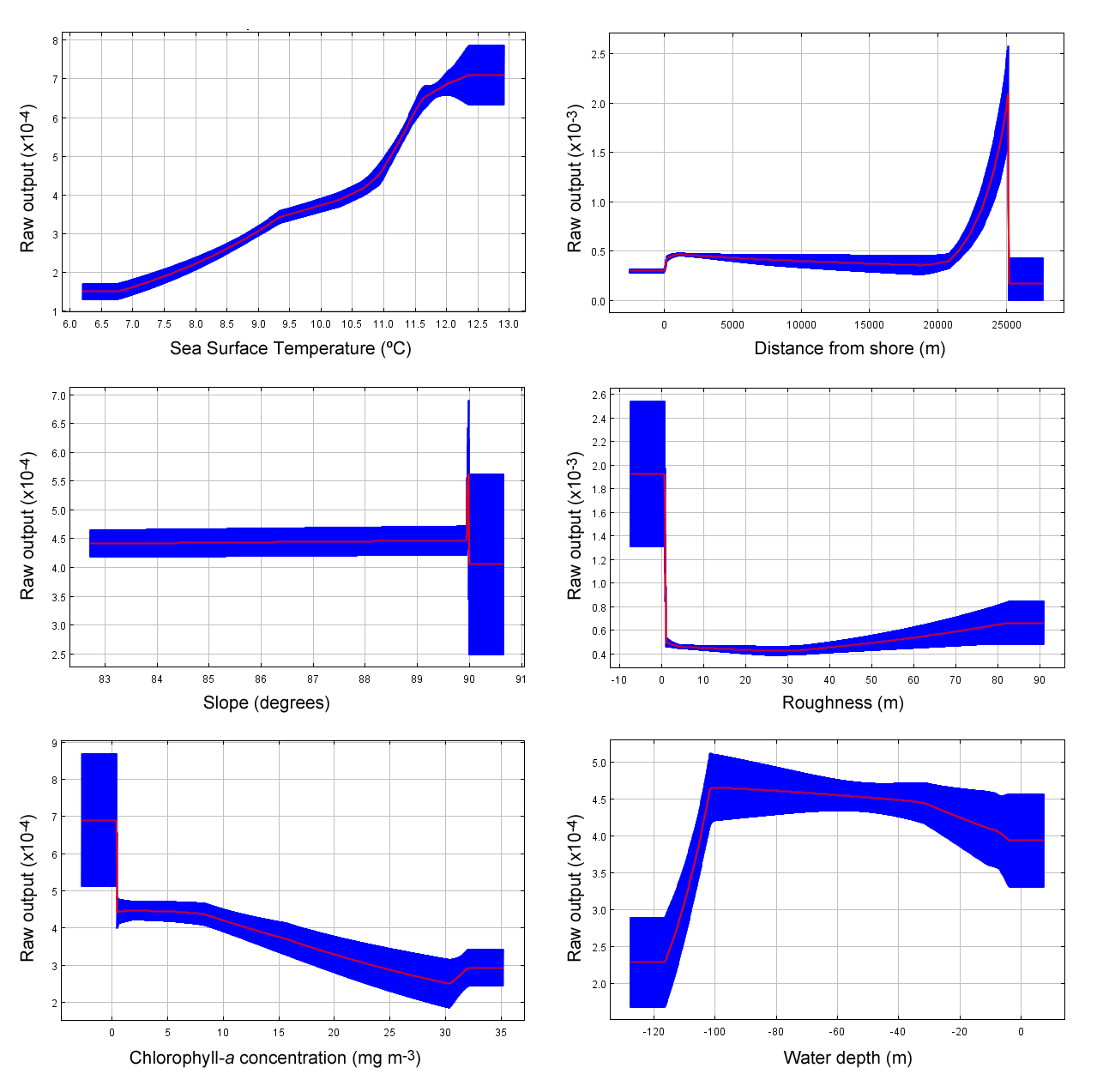

Supplement: S7 Fig — The curves show how the predicted relative occurrence rate changes as each PV is varied, keeping all other PVs at their average sample value. The curves represent the mean response of 20 replicate MaxEnt runs (red) and the mean +/- one standard deviation (blue, two shades for categorical variables). (TIF) [file pone.0244068.s007.tif]

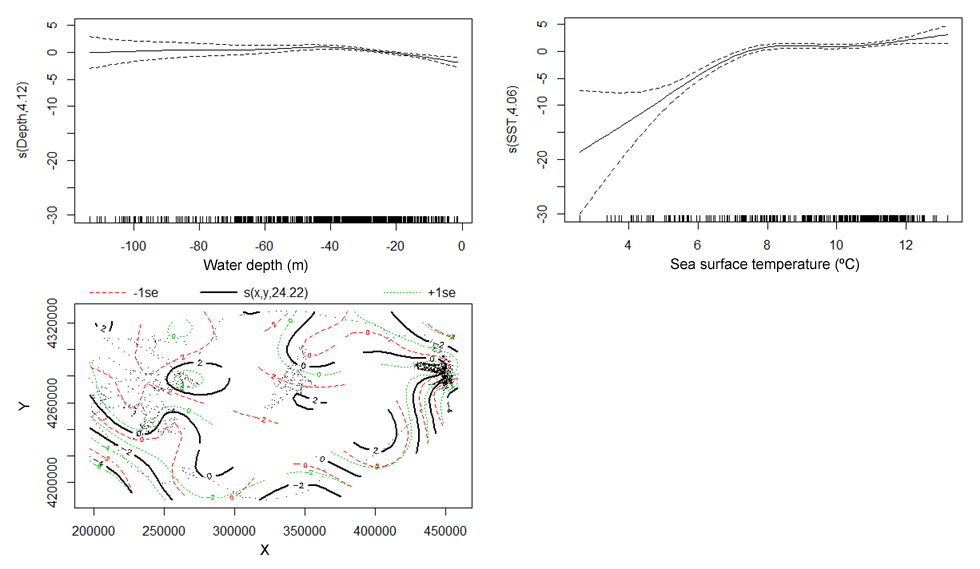

Supplement: S8 Fig — Dotted lines represent 95% confidence intervals. Degrees of freedom are show in parentheses on the y-axis label. The vertical lines above the x-axis show positions of the measured data points. (JPG) [file pone.0244068.s008.jpg]

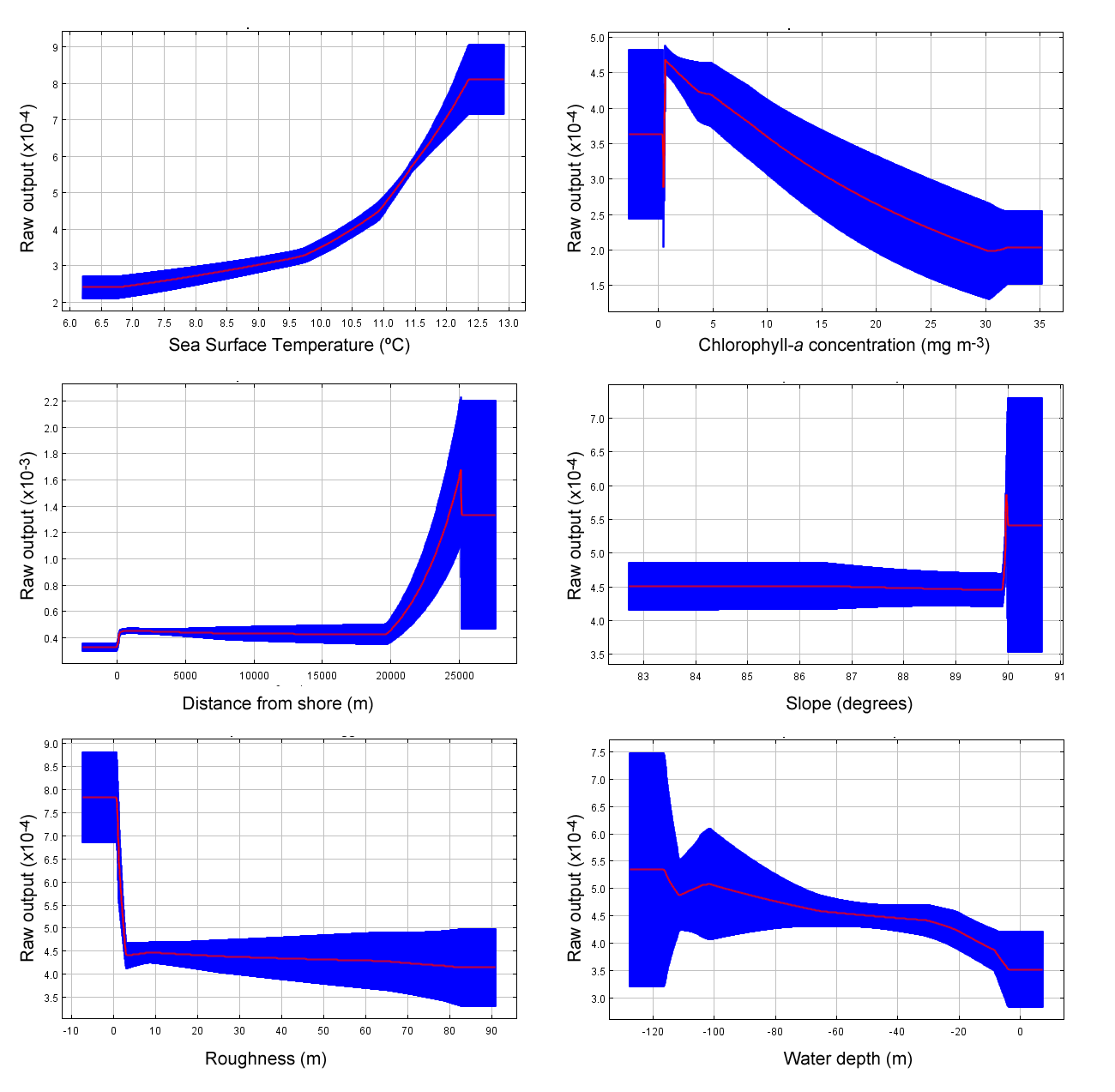

Supplement: S9 Fig — The curves show how the predicted relative occurrence rate changes as each PV is varied, keeping all other PVs at their average sample value. The curves represent the mean response of 20 replicate MaxEnt runs (red) and the mean +/- one standard deviation (blue, two shades for categorical variables). (TIF) [file pone.0244068.s009.tif]

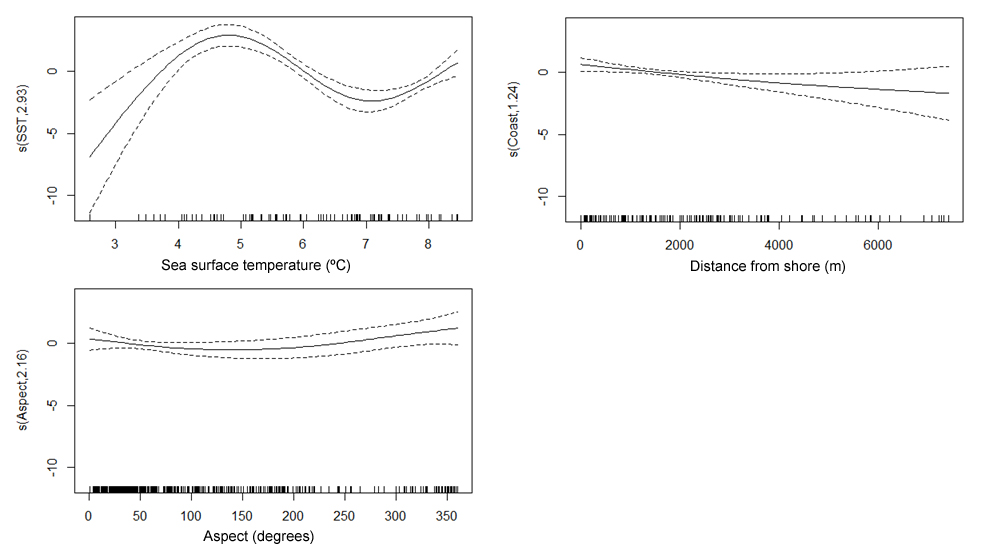

Supplement: S10 Fig — Dotted lines represent 95% confidence intervals. Degrees of freedom are show in parentheses on the y-axis label. The vertical lines above the x-axis show positions of the measured data points. (JPG) [file pone.0244068.s010.jpg]

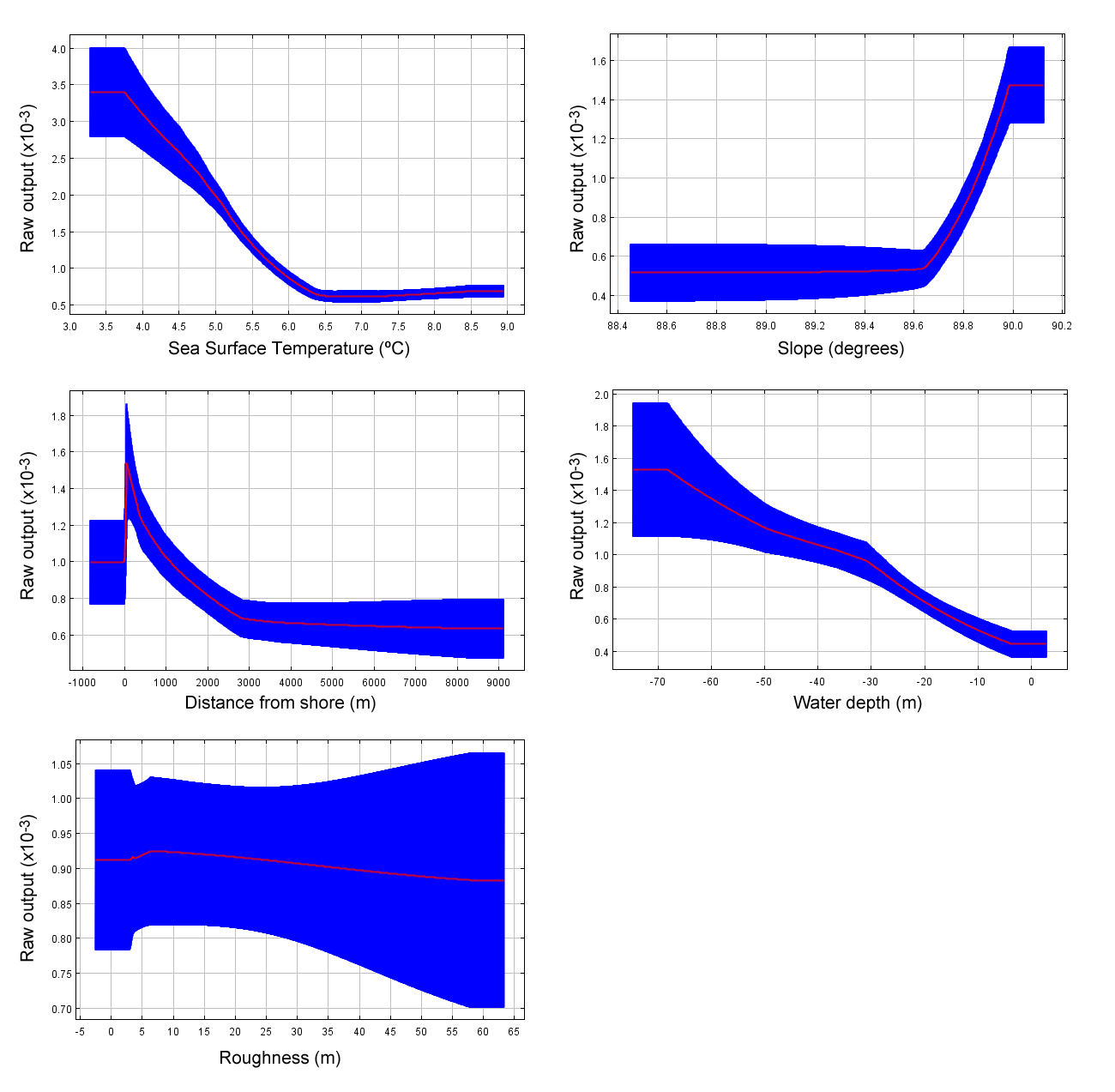

Supplement: S11 Fig — The curves show how the predicted relative occurrence rate changes as each PV is varied, keeping all other PVs at their average sample value. The curves represent the mean response of 20 replicate MaxEnt runs (red) and the mean +/- one standard deviation (blue, two shades for categorical variables). (TIF) [file pone.0244068.s011.tif]

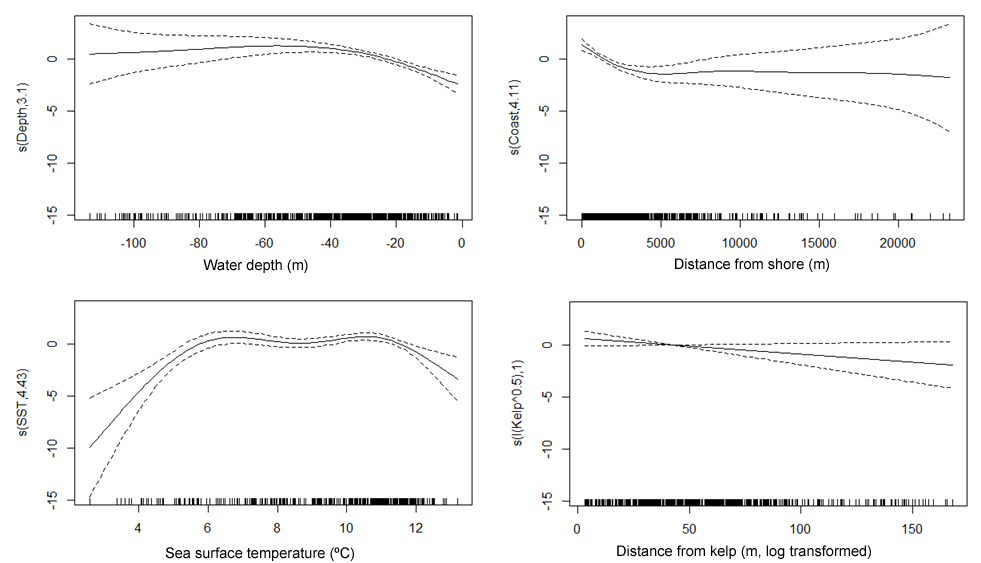

Supplement: S12 Fig — Dotted lines represent 95% confidence intervals. Degrees of freedom are show in parentheses on the y-axis label. The vertical lines above the x-axis show positions of the measured data points. (TIF) [file pone.0244068.s012.tif]

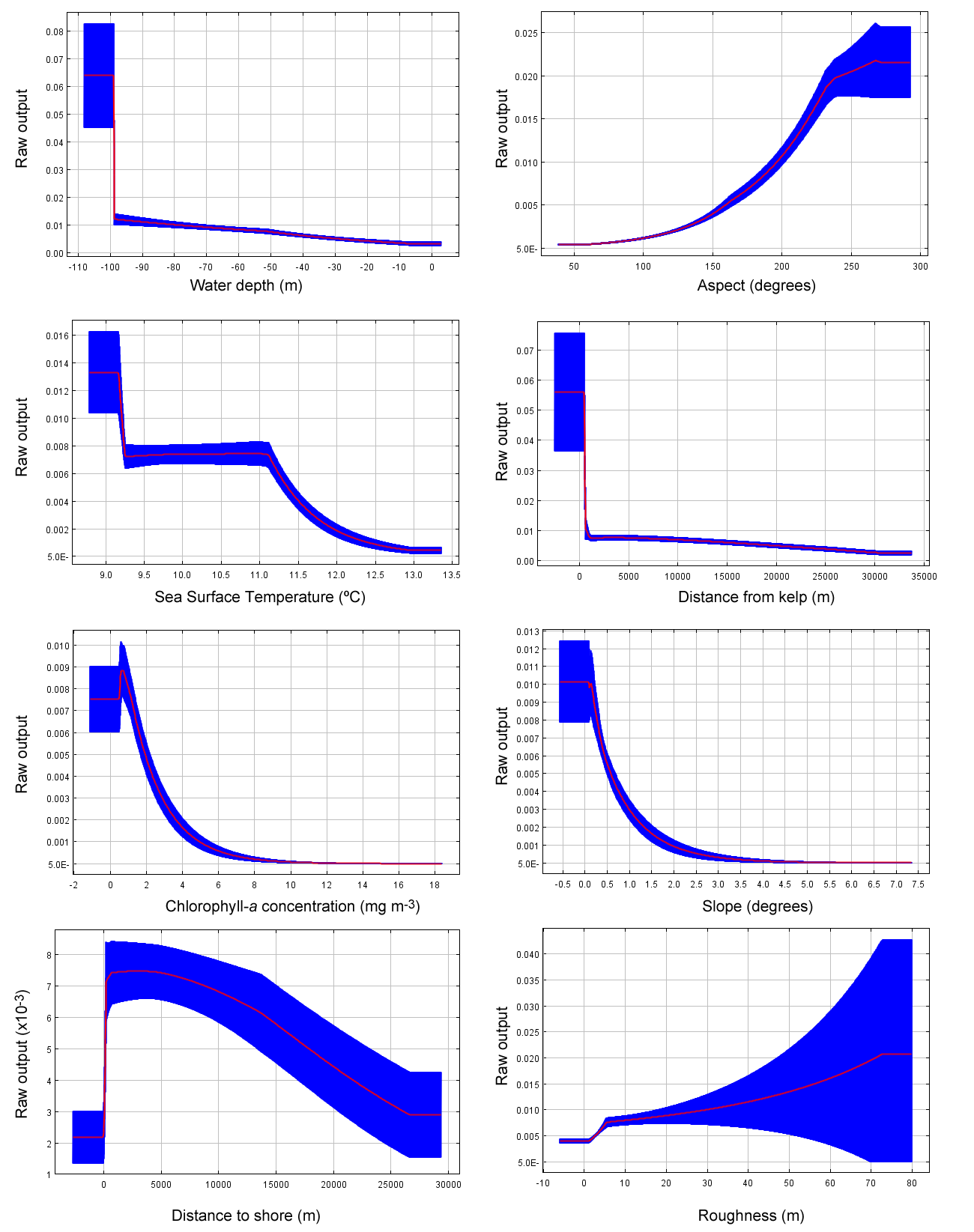

Supplement: S13 Fig — The curves show how the predicted relative occurrence rate changes as each PV is varied, keeping all other PVs at their average sample value. The curves represent the mean response of 20 replicate MaxEnt runs (red) and the mean +/- one standard deviation (blue, two shades for categorical variables). (TIF) [file pone.0244068.s013.tif]

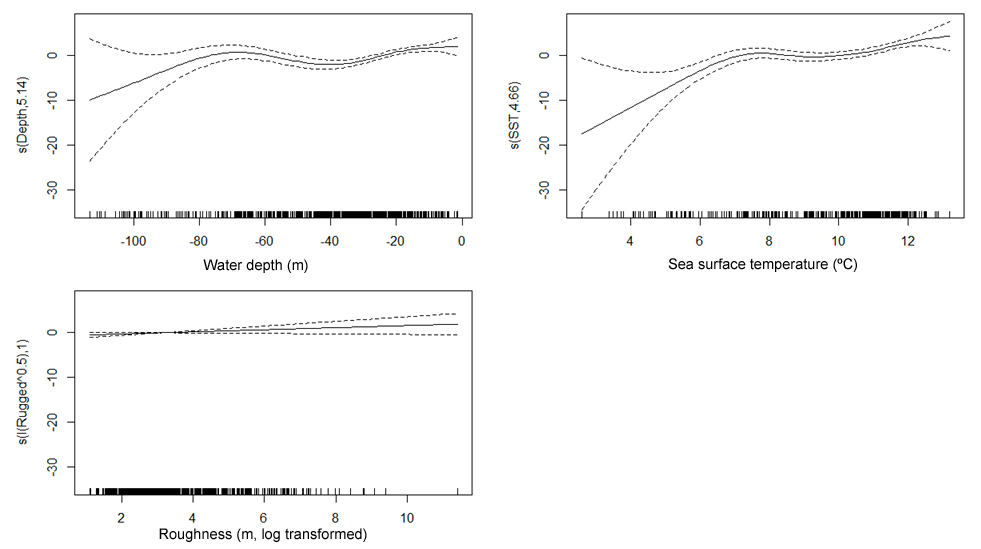

Supplement: S14 Fig — Dotted lines represent 95% confidence intervals. Degrees of freedom are show in parentheses on the y-axis label. The vertical lines above the x-axis show positions of the measured data points. (JPG) [file pone.0244068.s014.jpg]

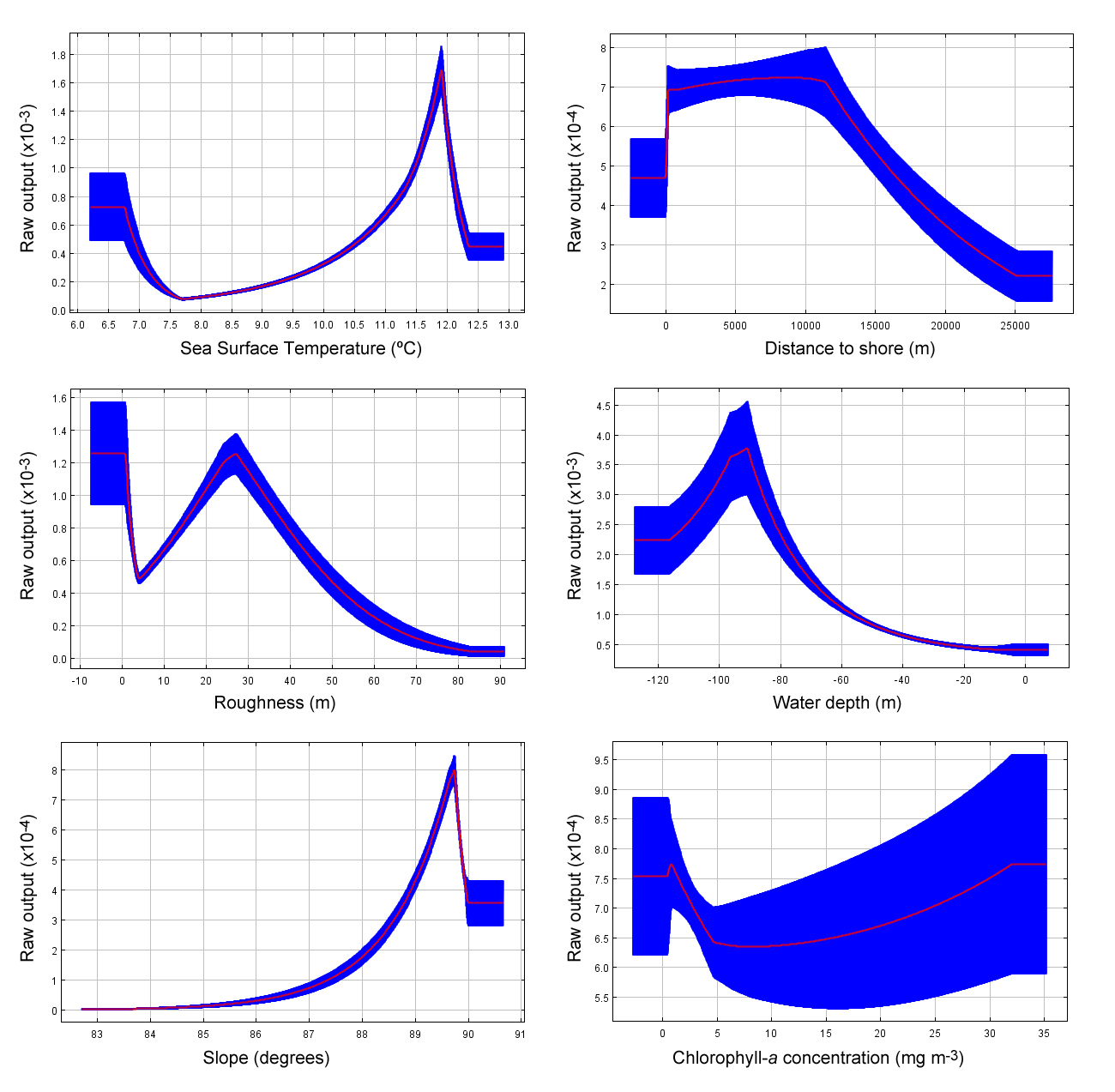

Supplement: S15 Fig — The curves show how the predicted relative occurrence rate changes as each PV is varied, keeping all other PVs at their average sample value. The curves represent the mean response of 20 replicate MaxEnt runs (red) and the mean +/- one standard deviation (blue, two shades for categorical variables). (TIF) [file pone.0244068.s015.tif]
